# Cori Ester as the Ligand for Monovalent Cations

**DOI:** 10.3390/molecules29092133

**Published:** 2024-05-04

**Authors:** Krystyna Stępniak, Tadeusz Lis, Elżbieta Łastawiecka, Anna E. Kozioł

**Affiliations:** 1 Faculty of Chemistry, Maria Curie-Skłodowska University, 20-031 Lublin, Poland; krstepniak@poczta.onet.pl (K.S.); elzbieta.lastawiecka@mail.umcs.pl (E.Ł.); 2 Faculty of Chemistry, University of Wrocław, 50-383 Wrocław, Poland; tadeusz.lis@uwr.edu.pl

**Keywords:** Cori ester, *α*-D-glucose-1-phosphate, sodium complex, potassium complex, ammonium complex, potassium sodium complex, ammonium sodium complex, crystal structure, NMR spectra

## Abstract

Gerty T. and Carl F. Cori discovered, during research on the metabolism of sugars in organisms, the important role of the phosphate ester of a simple sugar. Glucose molecules are released from glycogen—the glucose stored in the liver—in the presence of phosphates and enter the blood as α-D-glucose-1-phosphate (Glc-1PH_2_). Currently, the crystal structure of three phosphates, Glc-1PNa_2_·3.5·H_2_O, Glc-1PK_2_·2H_2_O, and Glc-1PHK, is known. Research has shown that reactions of Glc-1PH_2_ with carbonates produce new complexes with ammonium ions [Glc-1P(NH_4_)_2_·3H_2_O] and mixed complexes: potassium–sodium and ammonium–sodium [Glc-1P(X)_1.5_Na_0.5_·4H_2_O; X = K or NH_4_]. The crystallization of dicationic complexes has been carried out in aqueous systems containing equimolar amounts of cations (1:1; X–Na). It was found that the first fractions of crystalline complexes always had cations in the ratio 3/2:1/2. The second batch of crystals obtained from the remaining mother liquid consisted either of the previously studied Na+, K+ or NH_4_+ complexes, or it was a new sodium hydrate—Glc-1PNa_2_·5·H_2_O. The isolated ammonium–potassium complex shows an isomorphic cation substitution and a completely unique composition: Glc-1PH(NH_4_)_x_K_1−x_ (x = 0.67). The Glc-1P^2−^ ligand has chelating fragments and/or bridging atoms, and complexes containing one type of cation show different modes of coordinating oxygen atoms with cations. However, in the case of the potassium–sodium and ammonium–sodium structures, high structural similarities are observed. The 1D and 2D NMR spectra showed that the conformation of Glc-1P^2−^ is rigid in solution as in the solid state, where only rotations of the phosphate group around the C-O-P bonds are observed.

## 1. Introduction

Carbohydrates are a very important class of biological molecules. Commonly called sugars, they perform various functions in living organisms, from providing energy, through storing it, to building cellular structures. They are the only compounds that can be created in the process of synthesis from water, carbon dioxide, and chemical energy during photosynthesis; most of the compounds in this group are of plant origin. In the animal world, it is possible to synthesize sugars using, among other things, amino acids and lipids [1]. In the 19th century, physiologist Claude Bernard discovered glycogen, a polysaccharide composed of glucose residues connected by α-1,4-glycosidic bonds. It serves as a glucose reserve; when the body is in good condition, the liver stores excess sugar in the form of glycogen, while in states of hypoglycemia, it breaks down, and the released glucose molecules allow the normalization of sugar levels [2].

Gerty and Carl Cori studied glycogenolysis—the process of glycogen breakdown which, in the first stage, produces glucose-1-phosphate (called Cori ester) and a chain of sugar molecules shortened by one unit [3,4]. They determined the structure of the compound and also identified the enzyme that catalyzes its formation, i.e., phosphorylase [3]. During their research, they determined for the first time the essence of the function of carbohydrates in animal bodies, as well as the influence of insulin and adrenaline on the blood glucose level.

In the functioning of the body (with the exception of organic compounds), elements that regulate physiological functions are essential [5]. Such elements include water and a group of macro-elements: sodium, potassium, magnesium, and calcium. Their deficiency or excess causes disturbance of the alkaline–acid balance, heart problems, and muscle dysfunction. At the cellular level, Na ions play important roles in biological communication to trigger responses. The rapid inflow of sodium ions into the cell triggers the activation of neurons, which allows the transmission of nerve impulses [5]. The Na^+^ K^+^-ATPase, also called the sodium pump, is a cell membrane protein involved in the process of monovalent cation transport. This sodium–potassium pump actively transfers potassium into the cell, maintaining the correct cationic composition on both sides of the cell membrane. All animal cells remove Na ions and retain K. Potassium cations activate various intracellular processes, while sodium cations inside cells act as inhibitory factors [1].

A review of structural information in the CSD database [6] for crystals with the chemical composition K+Na+C+O+H revealed intriguingly little information about salts/complexes of this type. The phases examined so far containing these two cations are (a) carbonate NaK_2_(HCO_3_)CO_3_·2H_2_O [7]; (b) sodium potassium D-glucarate dihydrate [8]; (c) two citrates—sodium potassium hydrogen citrate and sodium dipotassium citrate [9,10]; and (d) the most intensively studied Rochelle salts—racemic trihydrate [11] and tetrahydrate [12] as well as enantiomeric potassium sodium (2*R*, 3*R*)-tartrate tetrahydrate, e.g., [13,14].

Moreover, the structure of any complex containing a phosphate ligand and K and Na cations has not been investigated.

Cori ester is a very important metabolite in animal organisms. Despite the significance of this ester, only a small number of structural characterizations of its complexes with monovalent cations of biological importance have been published so far. There are four papers presenting structural studies of the crystals of complexes with the *α*-D-glucose-1-phosphate anion. One sodium complex (Glc-1PNa_2_·3.5·H_2_O) [15,16] and two complexes containing potassium cations have been analyzed, i.e., Glc-1PK_2_·2H_2_O [17] and Glc-1PHK [18].

Considering the coexistence and competition of various molecules and ions in body fluids, we decided to expand this set of the Cori ester complexes. The synthesis and analysis of new complexes with ammonium cations and complexes containing two types of monovalent cations in the same phase were carried out. It was demonstrated over a hundred years ago that K and NH_4_ cations can exhibit the isomorphic substitution [19,20]. However, in the phases containing two other cations, e.g., K and Na, Na and NH_4_, the formation of complexes in which specific cations will have preferred coordination sites for the Glc-1P^2−^ anions can be expected. For this reason, we decided to synthesize and obtain single crystals of the ammonium salt of *α*-D-glucose phosphate and complexes containing two cations by utilizing Glc-1PH_2_ acid and carbonates. An X-ray structural analysis of single crystals and NMR spectroscopy of aqueous solutions were selected as the research methods.

## 2. Results and Discussion

### 2.1. Complexes in Solid Phase

**Figure 1:**
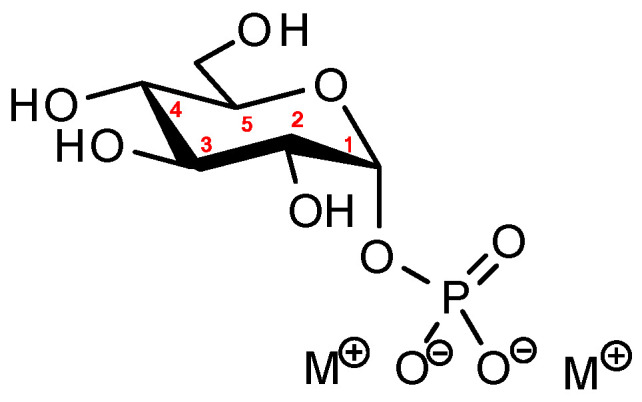
Structure of *α*-D-glucopyranosyl-1-phosphate ester.

The aim of the research presented here was to determine the role of the Cori ester (Figure 1) as a ligand coordinating monovalent cations (NH_4_, K and Na); this was possible to observe through obtaining crystalline complexes and identifying their structure (Tables S2–S8).

**Scheme 1:**
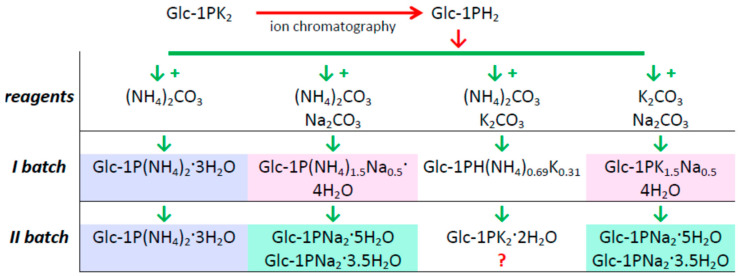
Crystalline products obtained after the reaction of *α*-D-glucose-1-phosphate ester with monovalent cations.

The first step of the synthesis was to obtain the Glc-1PH_2_ acid from the potassium complex through ion exchange chromatography. The acid subsequently reacted with selected carbonates to form complexes with various stoichiometries (Scheme 1). Crystals of these complexes were obtained by crystallization using the antisolvent method: propan-2-ol was added to the aqueous solution, followed by diffusion of ethanol vapors, which caused a supersaturation of the solution. The chemical composition of the obtained crystals was different and depended on the cations present in the system (Scheme 1). During the second crystallization step, the remaining mother liquor produced previously known hydrates of sodium and potassium complexes, except for the new sodium pentahydrate Glc-1P2Na-5H2O. Structural studies were performed for the resulting crystals, including previously known complexes, with detailed data presented in Table 1.

The reaction of Glc-1PH_2_ with ammonium carbonate gave the expected product (Table 2). However, the most interesting are the stoichiometries of complexes containing two cations: K+Na, NH_4_+Na and NH_4_+K. The first two products are obtained in the reaction according to the following scheme:

3 Glc-1P^2−^ + 3 Na^+^ + 3 **X**^+^ —> 2 Glc-1P**X**_1.5_Na_0.5_ + Glc-1PNa_2_ [**X**^+^ = K^+^, NH_4_^+^]

The Glc-1PK_1.5_Na_0.5_·4H_2_O and Glc-1P(NH_4_)_1.5_Na_0.5_·4H_2_O are isomorphic, but the ammonium–potassium complex has a completely different composition and structure (Scheme 1, Table 2). The products of a synthesis (crystallization) in the latter system are completely unpredictable, a mixture of complexes is formed, and the crystals are of poor quality and unstable. The first product formed in the crystallization process that was identified was the acidic complex with the NH_4_:K ratio of approximately 70%:30% [Glc-1PH(NH_4_)_0.69_K_0.31_]. Both cations occupy the same position in the crystal lattice, i.e., the isomorphic substitution occurs. Only the already known potassium complex, Glc-1PK_2_·2H_2_O, was identified in the second crystallization, while the remaining products were amorphous, which was confirmed by X-ray microdiffraction patterns.

**Figure 2:**
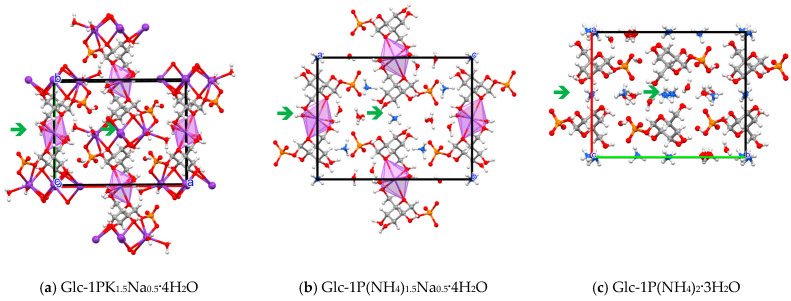
The crystal packing of isomorphic complexes; view down the c axis. The Na/NH_4_ cation positions on the 2-fold axes are marked as 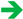.

The lattice parameters, the symmetry of crystals, and the positions of the cations in the unit cell indicate that the Glc-1PKNa, Glc-1P(NH4)Na and Glc-1P2(NH4) complexes are isomorphic (Table 1 and Table 2). They all crystallize in the *P* 2_1_2_1_2 space group, and the two (of three) positions occupied by the cations lie on the two-fold symmetry axes (Figure 2). Research on this group of complexes revealed another interesting feature. Namely, the ammonium complex Glc-1P2(NH4) crystals are hygroscopic and stable for 1–2 months. However, the co-presence of small amounts of Na or K cations in the crystal lattice stabilizes the crystals, which do not decompose even after ten years.

In the crystals of Glc-1PKNa and Glc-1P(NH_4_)Na, the sodium cations have coordinates ½, 0, z, while the K/NH_4_ cations occupy positions x, y, z and ½, ½, z (Figure 2a,b). Due to this arrangement of atomic positions, the stoichiometry of cations K/NH_4_–Na in the crystal is 3/2:1/2, whereas the three ammonium cations in the Glc-1P2(NH4) complex (Figure 2c) are located in the general position on the two-fold axis (the Wyckoff position b) and are disordered around the second position of the two-fold axis (the Wyckoff position a) [21].

**Figure 3:**
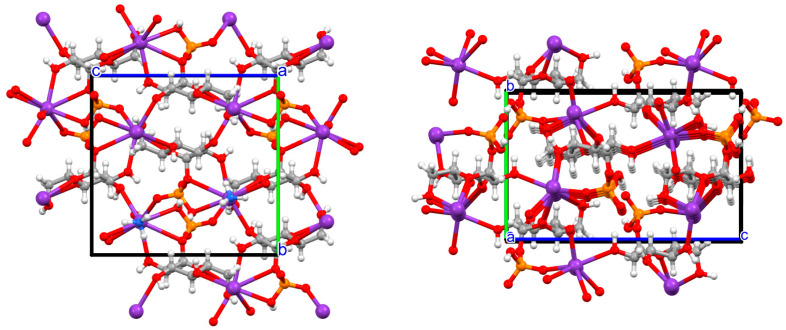
The crystal packing of α-D-glucose 1-hydrogenphosphate complexes, viewed the a axis. (**left**) Glc-1PH(NH_4_)_0.69_K_0.31_; (**right**) Glc-1PHK [18].

Some structural similarities can also be observed in the anhydrous structures of hydrogen phosphates, Glc-1PH(NH4)K and Glc-1PHK: the symmetry of the crystal lattice, the translation along a, the volume of the unit cell, and the anion orientation are preserved (Table 2, Figure 3). However, the isomorphic substitution of the K^+^ cations with 69% of NH_4_^+^ cations changed the lengths of translations b and c. This is due to replacing most of the K-O coordination bonds with the N-H–O hydrogen bonds.

### 2.2. Structure of α-D-Glucopyranosyl-1-Phosphate Dianion

**Figure 4:**
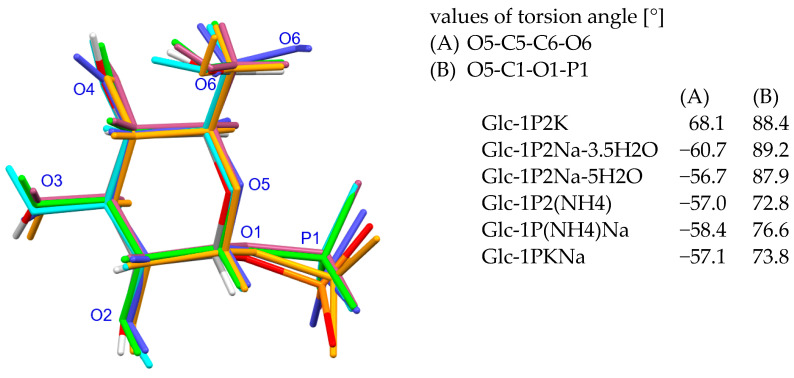
Molecular fitting of Glc-1P^2−^ dianions; the pyranose ring is fitted. The Glc-1P2K anion is marked in navy blue, Glc-1P2Na-3.5H2O in yellow, Glc-1P2Na-5H2O in standard atom colors, Glc-1P2(NH4) in green, Glc-1P(NH4)Na in light blue, and Glc-1PKNa in burgundy. Values of the torsion angles describing orientation of the O6 hydroxyl and P1 phosphate atoms are given.

The Cori ester, α-D-glucopyranose-1-phosphate, is composed of a pyranose ring substituted with the phosphate group attached to its C1 position, three hydroxyl groups (the C2, C3, C4 positions), and the hydroxymethylene group (the C5 position). The ring adopts the chair conformation, which is not significantly changed by interactions with cations and water molecules present in the crystals. Fitting of the pyranose rings of all dianions observed in the studied crystals shows that the equatorial atoms directly connected to this ring (O1, O2, O3, O4, C6) have a rigid orientation of C-O/C bonds (Figure 4). A detailed comparison of dianion conformations shows that the differences in the position of the carbon atoms are <0.2 Å, and the position of the P1 atom differs by a maximum of 0.5 Å (Glc-1P2Na-5H2O vs. Glc-1PKNa).

Different coordination modes of cations and hydrogen bonds formed between anions and water molecules cause variable directions of the O-H bond vectors of the O3 and O4 hydroxyl groups, while the O2-H group has a rigid orientation (as shown in Figure 4). The conformations around the C5-C6 bond are well described by the O5-C5-C6-O6 torsion angle. In all complexes containing Na and NH_4_ cations, the orientation of the O6-H hydroxyl group relative to the O5 pyranose atom is -*synclinal*; only in the dipotassium complex does it adopt a +*synclinal* conformation (Figure 4). Some differences are also noticeable in the relative position of the pyranose O5 oxygen atom and P1 phosphorus. The torsion angles of the *synclinal* orientation in the O5-C1-O1-P1 fragment differ by approximately 10–15°; this angle adopts higher values (≈90°) in the homocationic complexes and smaller values (≈75°) in heterocationic ones (Figure 4).

### 2.3. Environment of α-D-Glucopyranosyl-1-Phosphate Dianion

**Figure 5:**
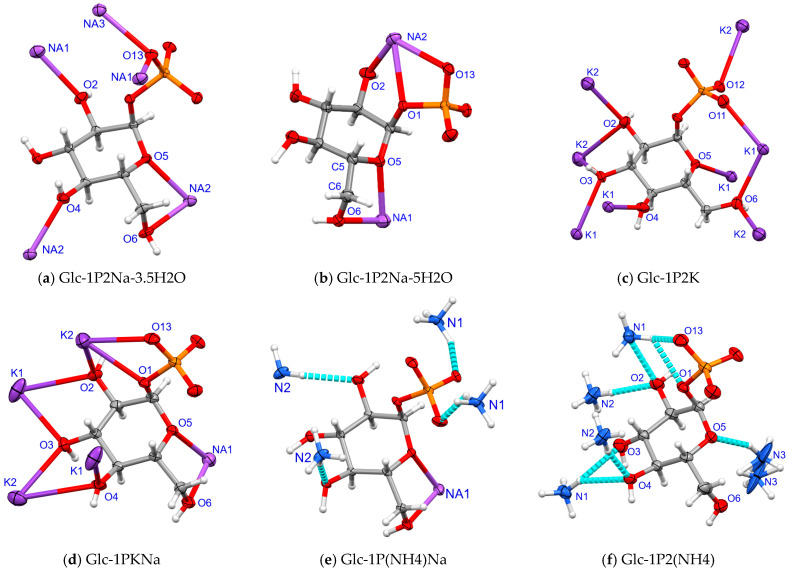
View of the Glc-1P2- dianions and surrounding cations. The hydrogen bonds are marked as blue dahsed lines. The intra-anionic distances between chelating O5 and O6 atoms are as follows: (**a**) 2.75 Å; (**b**) 2.76 Å; (**c**) 2.91 Å; (**d**) 2.77 Å; (**e**) 2.80 Å; and (**f**) 2.76 Å.

The analysis of the cation coordination by the Glc-1P dianion shows significant differences in the mode of interactions (Figure 5). Anions are multidentate ligands; they can potentially coordinate cations through the nine oxygen atoms present in their structures. Additionally, the cation coordination spheres are completed by water molecules present in the crystals (Tables S10–S14). The Supplementary Materials contain information about the coordination numbers of cations, the values of the X^+^–O cation coordination bonds, and the most important hydrogen bonds.

The simplest coordination mode is observed in the structure of the Glc-1P2Na-5H2O complex (Figure 5b, Table S12). Five oxygen atoms are involved in coordination, with the O5 and O6 oxygen atoms performing chelating functions and the O1, O2, O13 oxygen atoms being the second chelating fragment. In the dipotassium dihydrate complex (Figure 5c, Table S9), the anion is surrounded by eight cations, seven oxygen atoms are involved in the coordination, and two chelating fragments can be observed here. The cation–anion interactions in Glc-1P2(NH4) occur through N-H–O hydrogen bonds (Table S16, Figure 5f).

**Figure 6:**
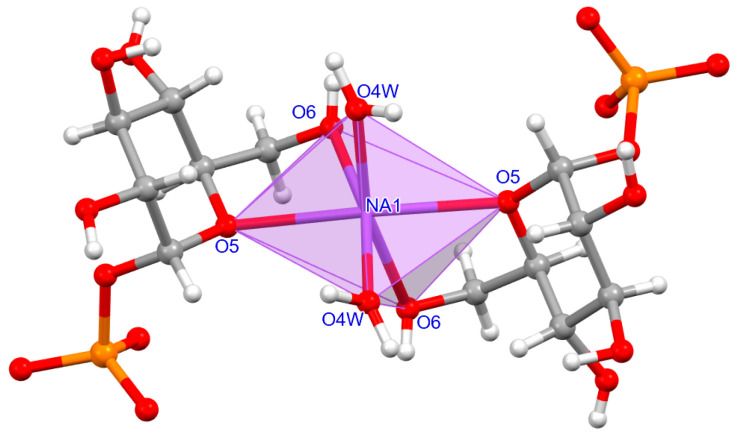
View of two Glc-1P^2−^ dianions chelating the Na cation located on the two-fold axis.

It seems that the difference in the ionic radii of Na (1.14–1.16 Å) and K (1.52–1.65 Å) [22] favors the selective binding of sodium to the chelating fragment O5-C5-C6-O6. The intra-anionic O5 to O6 distance in Na complexes is slightly smaller than in Glc-1P2K (where there is no chelation) and is equal to 2.75–2.8 Å (Figure 5). The most interesting observation comes from analyzing crystal structures containing two cations, i.e., K/Na and NH_4_/Na. In addition to their isomorphic nature, which was discussed earlier, the mode in which the sodium cation interacts with the anion is identical to that in which the disodium hydrates. In each of these structures, the Na cation is chelated by the O5 and O6 atoms and, if the symmetry of the crystal allows it, these cations are positioned on the two-fold symmetry axis (Figure 5a,b,d,e and Tables S11–S14). This position of the cation and chelation by the anions creates a dimeric anion–Na–anion structure (Figure 6).

### 2.4. Environment of α-D-Glucopyranosyl-1-Hydrogenphosphate Anion

**Figure 7:**
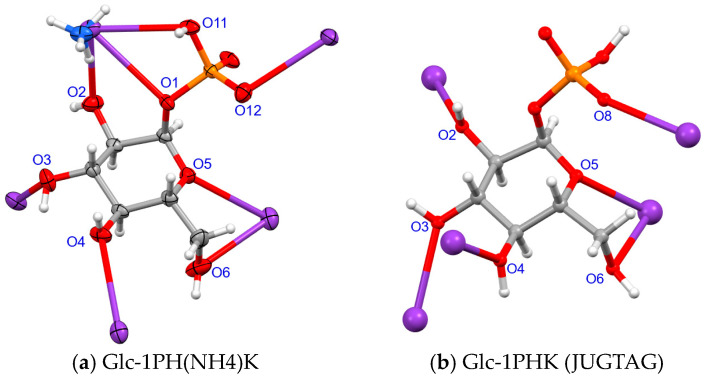
View of the Glc-1HP^−^ anions and surrounding cations. In the structure of the NH_4_/K complex (**a**), the isomorphic cation substitution (NH_4_:K = 69%:31%) is marked only in one position.

Previously, the similarities in the structure of the crystal network of the anhydrous hydrogen phosphate complexes, i.e., Glc-1PHK and Glc-1PH(NH4)K, were discussed. The analysis of the potassium mode of coordination and isomorphically substituted NH_4_/K ions shows even greater convergence. The surroundings of the anions are similar except for the mode of coordination to the phosphate group (Figure 7). In the ammonium–potassium complex, NH_4_/K cations are chelated by three oxygen atoms: O11 and O1 of the phosphate and O2 of the hydroxyl group. Meanwhile, in the potassium complex, only the K-O2 bond is formed. As a result, the potassium coordination number changes from 6 to 8 in Glc-1PH(NH4)K crystal (Table S15).

### 2.5. Nuclear Magnetic Resonance Studies

**Figure 8:**
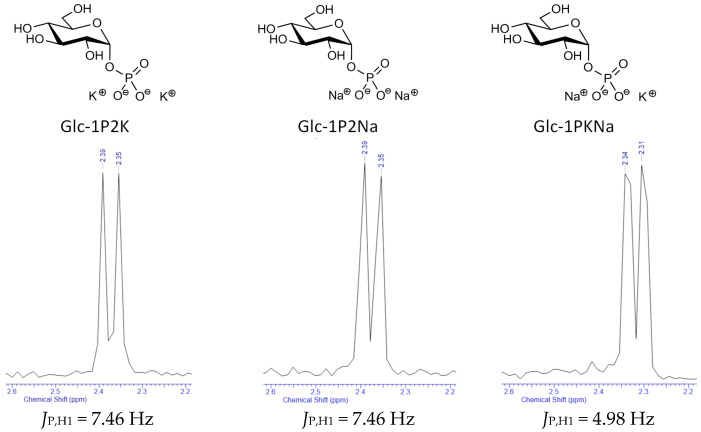
^31^P NMR spectra of α-D-glucose 1-phosphate complexes (Glc-1P2K, Glc-1P2Na and Glc-1PKNa) in D_2_O, with no decoupling, at 202 MHz.

NMR spectroscopy in aqueous solution was used to study the structure of α-D-glucose-1-phosphate anion of the three selected complexes: Glc-1P2K, Glc-1P2Na, and Glc-1PKNa. The analysis of ^1^H NMR, ^13^C NMR, and ^31^P NMR spectra showed that their profiles had striking similarities. They exhibited consistent shapes and identical coupling constants, which can be seen in Figures S1–S21 in the Supplementary Materials. The signal originating from the anomeric proton (H1) close to the phosphate group showed minor variations within the experimental error. However, also notable is the change in the coupling constant for H_6a_ and H_6b_ (Table S1), which may be caused by possible chelation to the O5, O6 atoms. The ^31^P NMR experiments without proton decoupling confirmed the coupling of the anomeric proton to the phosphorus atom of the phosphate group. The singlet signal undergoes a splitting process that results in a doublet signal. The characteristic splitting from protons coupled with P in each compound can be seen in Figure 8.

The measured {^31^P}–{^1^H} coupling constant for both homocationic complexes, Glc-1P2K and Glc-1P2Na, was 7.46 Hz. The measured coupling constant aligns with that previously observed for α-D-glucose 1-phosphate disodium (Glc-1P2Na) [23]. However, the measured coupling constant was one-third lower, at 4.98 Hz, in the case of the heterocationic Glc-1PKNa. The detection of such a large change in the {^31^P}–{^1^H} coupling constant within the mixed phosphate complex Glc-1PKNa is interesting and suggests that it is most likely the result of a change in conformation resulting from the interaction of two different metal cations with the phosphate group.

## 3. Materials and Methods

### 3.1. General Information

The reagents were obtained from commercial suppliers and used without further purification. Dipotassium glucose-1-phosphate dihydrate (Glc-1PK_2_·2H_2_O 96%) was purchased from Reanal (Budapest, Hungary), Amberlit IR-120 was purchased from Aldrich (Milwaukee, WI, USA); disodium carbonate, dipotassium carbonate, diammonium carbonate, HCl 2n, ethanol 96%, and propan-2-ol were purchased from POCH S.A (Gliwice, Poland). pH monitoring was performed using an Elmetron CP-315m pH-meter (Zabrze, Poland). Elemental analyses were performed on a PerkinElmer CHN 2400 analyzer (USA). 

The NMR spectra were recorded using a Bruker Ascend spectrometer (^1^H 500 MHz, ^13^C NMR 126 MHz, ^31^P NMR 202 MHz) with D_2_O as the solvent, with a sample molarity of 0.05 mmol/mL at room temperature. Chemical shifts (δ) are given in ppm relative to residual H_2_O (^1^H) as a reference. Coupling constants (J) are in Hz. The following abbreviations of signal patterns are as follows: s (singlet), d (doublet), t (triplet), q (quartet), m (multiplet), br (broad).

The single crystal diffraction data were collected at room temperature with a SuperNova diffractometer (Oxford Diffraction; Agilent, Yarnton, UK [24]) with the graphite monochromated CuKα radiation (λ = 1.54184 Å), except for the unstable Glc-1P2(NH4) complex, for which the data collection was performed on a KM4 diffractometer with Mo Ka radiation (λ = 0.71069 Å). A CrysAlisPro 1.171.42.79a program system [25] or KM4 Software ver. KM4B8 (Wrocław, Poland) [26] was used for data collection, cell refinement, and data reduction. The intensities were corrected for Lorentz and polarization effects, and multi-scan absorption corrections were applied. The crystal structure was solved by direct methods using the SHELXT program and refined by the full-matrix least squares method on F^2^ using the SHELXL-2018/3 program [27,28]. The non-hydrogen atoms were refined with anisotropic displacement parameters, The carbon-bonded H-atoms were positioned at calculated positions and refined using the riding model. Hydrogen atoms bonded to oxygen atoms (in OH groups, water molecules) were treated depending on the quality of the crystal and the diffraction data. Some of them were located from differential electron density maps, some in calculated positions, and then the positions were refined or fixed. The experimental details and final atomic parameters for the analyzed crystals were deposited with the Cambridge Crystallographic Data Centre as a Supplementary Material (CCDC Nos 2345573-2345579).

#### 3.1.1. Syntheses

Glc-1PK_2_ was subjected to ion exchange chromatography to obtain acid (Glc-1PH_2_). The potassium complex (0.194 g; 0.5 mmol) was dissolved in 25 mL of redistilled water and applied to an Amberlit IR-120 loaded column (45 cm × 2 cm). Elution with water from a column yielded 28 mL of solution with a pH of 3.25. The aqueous Glc-1PH_2_ solution was rotary-evaporated to 22 mL.

##### Preparation of the Glc-1P2(NH_4_) Complex

While stirring, 0.048 g (0.5 mmol) of (NH_4_)_2_CO_3_ was added to 17 mL of Glc-1PH_2_ solution. The resulting mixture had a pH of 5.96. Then, 19 mL of propan-2-ol was added to the solution, obtaining an opalescent solution with a pH of 8.01. The solution was crystallized using an antisolvent vapor diffusion method. For this purpose, a vessel with the solution was placed in a chamber containing ethanol. Crystallization was carried out at a temperature of 10 °C; the first crystals appeared after 24 h. After eight days, the solution from above the crystals was pipetted and the crystals were subjected to diffraction studies, with a yield of 55%. The mother liquor left after a crystallization was further crystallized.

##### Preparation of the Glc-1PKNa Complex

While stirring, 0.035 g (0.25 mmol) of K_2_CO_3_ and 0.026 g (0.25 mmol) of Na_2_CO_3_ were added to 10 mL of Glc-1PH_2_ solution (0.25 mmol), and the resulting mixture had a pH of 6.23. Next, to induce precipitation, 28 mL of propan-2-ol was added to the solution, obtaining an opalescent solution with a pH of 7.89. The solution was crystallized using an antisolvent vapor diffusion method. For this purpose, the beaker with the solution was placed in a chamber containing ethanol. Crystallization occurred at room temperature; the first crystals appeared after 24 h. After two weeks, the solution was pipetted from above the crystals. Crystals with a rod morphology were subjected to diffraction measurements, with a yield of 60%. The mother liquor left after crystallization (pH = 8.11) was further crystallized, and after 20 days, a second batch of crystals was obtained.

##### Preparation of the Glc-1P (NH_4_)Na and Glc-1P (NH_4_)K Complexes

To obtain these complexes, an analogous procedure was used as for the synthesis of the Glc-1PNaK mixed complex.

The first crystal fraction of the Glc-1PKNa and Glc-1P(NH_4_)Na samples contained crystals with a stoichiometry of Glc-1PK_1.5_Na_0.5_·4H_2_O and Glc-1P(NH_4_)_1.5_Na_0.5_·4H_2_O. The second batches contained three types of crystalline phases. The previously mentioned complexes were present in small amounts, while the majority were crystals of the sodium complex with various degrees of hydration, being either a trihemihydrate or a new pentahydrate.

Elemental analyses were performed for complexes selected for NMR studies:

Glc-1PK_2_·2 H_2_O Calcd C 19.35%, H 4.06%; found C 19.31%, H 4.08%;

Glc-1PNa_2_·5 H_2_O Calcd C 18.28%, H 5.37%; found C 18.09%, H 5.29%;

Glc-1PK_1.5_Na_0.5_·4 H_2_O Calcd C 18.00%, H 4.78%; found C 18.18%, H 4.80%.

#### 3.1.2. NMR Data of α-D-Glucose 1-Phosphate Complexes

##### Dipotassium α-D-Glucose 1-Phosphate (Glc-1P2K)

^1^H NMR (500 MHz, D_2_O) δ ppm 3.28 (dd, J = 10.09, 9.14 Hz, 1 H, H_4_), 3.37 (ddd, J = 9.62, 3.47, 1.73 Hz, 1 H, H_2_), 3.63 (dd, J = 12.30, 5.04 Hz, 1 H, H_6a_), 3.68 (t, J = 9.46 Hz, 1 H, H_3_), 3.77 (dd, J = 12.14, 2.36 Hz, 1 H, H_6b_), 3.82 (ddd, J = 10.17, 5.12, 2.36 Hz, 1 H, H_5_), 5.34 (dd, J = 7.57, 3.47 Hz, 1 H, H_1_); ^13^C NMR (126 MHz, D_2_O) δ ppm 60.71 (s), 69.76 (s), 71.91 (s), 72.25 (d, J = 6.36 Hz), 73.16 (s), 93.51 (d, J = 5.45 Hz); ^31^P{^1^H} NMR (202 MHz, D_2_O) δ ppm 2.39; ^31^P NMR (202 MHz, D_2_O) δ ppm 2.37 (d, J = 7.46 Hz).

##### Disodium α-D-Glucose 1-Phosphate (Glc-1P2Na)

^1^H NMR (500 MHz, D_2_O) δ ppm 3.28 (dd, J = 10.25, 9.30 Hz, 1 H, H_4_), 3.36 (ddd, J = 9.77, 3.47, 1.89 Hz, 1 H, H_2_), 3.63 (dd, J = 12.30, 5.04 Hz, 1 H, H_6a_), 3.68 (t, J = 9.46 Hz, 1 H, H_3_), 3.77 (dd, J = 12.30, 2.52 Hz, 1 H, H_6b_), 3.82 (ddd, J = 10.01, 5.28, 2.36 Hz, 1 H, H_5_), 5.34 (dd, J = 7.57, 3.47 Hz, 1 H, H_1_); ^13^C NMR (126 MHz, D_2_O) δ ppm 60.70 (s), 69.76 (s), 71.91 (s), 72.25 (d, J = 7.27 Hz), 73.16 (s), 93.51 (d, J = 5.45 Hz); ^1^P{^1^H} NMR (202 MHz, D_2_O) δ ppm 2.35; ^31^P NMR (202 MHz, D_2_O) δ ppm 2.37 (d, J = 7.46 Hz). 

##### Potassium Sodium α-D-Glucose 1-Phosphate (Glc-1PKNa)

^1^H NMR (500 MHz, D_2_O) δ ppm 3.29 (t, J = 9.62 Hz, 1 H, H_4_), 3.37 (ddd, J = 9.77, 3.47, 1.58 Hz, 1 H, H_2_), 3.64 (dd, J = 12.30, 5.36 Hz, 1 H, H_6a_), 3.68 (t, J = 9.50 Hz, 1 H, H_3_), 3.77 (dd, J = 12.30, 2.60 Hz, 1 H, H_6b_), 3.82 (ddd, J = 10.17, 5.12, 2.36 Hz, 1 H, H_6_), 5.35 (dd, J = 7.57, 3.47 Hz, 1 H, H_1_); ^13^C NMR (126 MHz, D_2_O) δ ppm 60.69 (s), 69.74 (s), 71.91 (s), 72.23 (d, J = 6.36 Hz), 73.14 (s), 93.53 (d, J = 4.54 Hz); ^1^P{^1^H} NMR (202 MHz, D_2_O) δ ppm 2.33; ^31^P NMR (202 MHz, D_2_O) δ ppm 2.32 (d, J = 4.98 Hz).

## 4. Conclusions

Two new complexes of Cori ester (Glc-1PH_2_) with Na and NH_4_ cations were synthesized, and three complexes containing two different monovalent cations in their structure were obtained: Na+K, NH_4_+Na, and NH_4_+K. The structures of these five complexes were compared with previously published data for three other complexes, viz. Glc-1PNa_2_·3.5·H_2_O, Glc-1PK_2_·2H_2_O and Glc-1PHK. All the crystal lattices have non-centrosymmetric space groups: *P*2_1_2_1_2, *P*2_1_2_1_2_1_, *P*2_1_ and *C*2.

The conformation of the α-D-glucose-1-phosphate anion is essentially rigid. However, small changes in the directions of the hydroxyl O-H bonds and the position of the phosphate group are possible. The torsion angle O5-C1-O1-P1, indicating the relative position of the pyranose O5 and phosphate P1 atoms, adopts higher values (≈90°) in the homocationic complexes and smaller values (≈75°) in heterocationic ones. The ^31^P NMR spectra indicate that the anion in aqueous solutions may undergo similar conformational changes depending on the type of cations present, such as K, Na, or a mixture of Na and K.

The most important conclusions result from observations of the cation coordination modes of the Glc-1P^2−^ and Glc-1PH^−^ anions. If sodium cations are included in the mono- or dication complexes, they are chelated by the same cation oxygen atoms (O5 and O6). In the crystal, this Na cation is usually located on the two-fold symmetry axis, giving a cation–anion stoichiometry of 0.5:2. As a result, the remaining charge of the anion is neutralized by the second cation (K^+^ or NH_4_^+^) in a molar amount of 1.5, and complexes have the formula Glc-1P(X)_1.5_Na_0.5_·4H_2_O. Excess sodium cations form sodium complexes in the second crystallization step. Moreover, the same position is occupied by Na in the crystal net of sodium α-D-glucose-1-phosphate trihemihydrate and pentahydrate.

Conversely, it is neither straightforward nor clear how we might obtain a crystalline complex in which, as would be expected, the K^+^ and NH_4_^+^ cations replace one another isomorphically. The reaction products did not crystallize well, and the isolated crystal was anhydrous, like the previously identified Glc-1-PHK, and had the composition Glc-1-PH(NH_4_)_0.69_K_0.31_.

By investigating the complexes of the Cori ester (Glc-1PH_2_) with additional monovalent cations, we will expand on the findings presented above.

## Figures and Tables

**Table 1:** Crystallographic data for potassium/sodium Glc-1P complexes.

Identification Code	Glc-1PKNa	Glc-1P2Na-5H2O	Glc-1P2Na-3.5H2O *	Glc-1P2K *
Formula	Glc-1PK_1.5_Na_0.5_·4H_2_O	Glc-1PNa_2_·5·H_2_O	Glc-1PNa_2_·3.5·H_2_O	Glc-1PK_2_·2H_2_O
Formula weight	400.33	394.18	367.15	372.35
Crystal system	orthorhombic	orthorhombic	monoclinic	monoclinic
Space group	*P* 2_1_2_1_2	*P* 2_1_2_1_2_1_	*C* 2	*P* 2_1_
Cell parameters	a = 16.8360(4) Å	a = 6.6347(5) Å	a = 8.4325(2) Å	a = 7.5234(3) Å
	b = 13.4089(3) Å	b = 8.7585(9) Å	b = 10.1983(2) Å	b = 9.0643(3) Å
	c = 6.6729(1) Å	c = 27.129(2) Å	c = 16.5857(4) Å	c = 10.4588(4) Å
	β = 90°	β = 90°	β = 99.147(2)°	β = 110.576(5)°
Volume	1506.42(5) Å^3^	1576.5(2) Å^3^	1408.19(6) Å^3^	667.73(5) Å^3^
Final R indices [I > 2σ(I)]	R1 = 0.0321 wR2 = 0.0852	R1 = 0.0277 wR2 = 0.0613	R1 = 0.0243 wR2 = 0.0635	R1 = 0.0294 wR2 = 0.0774
CCDC No	2345577	2345578	2345575	2345574

* redetermination of structure [16,17].

**Table 2:** Crystallographic data for ammonium/potassium/sodium Glc-1P complexes.

Identification Code	Glc-1P2(NH4)	Glc-1P(NH4)Na	Glc-1PH(NH4)K	Glc-1PHK [18]
Formula	Glc-1P(NH_4_)_2_·3H_2_O	Glc-1P(NH_4_)_1.5_Na_0.5_· 4H_2_O	Glc-1PH(NH_4_)_0.69_K_0.31_	Glc-1PHK
Formula weight	348.25	368.74	283.70	298.23
Crystal system	orthorhombic	orthorhombic	orthorhombic	orthorhombic
Space group	*P* 2_1_2_1_2	*P* 2_1_2_1_2	*P* 2_1_2_1_2_1_	*P* 2_1_2_1_2_1_
Cell parameters	a = 13.756(7) Å	a = 16.9272(3) Å	a = 7.147(4) Å	a = 7.35(4) Å
	b = 16.929(7) Å	b = 13.3171(3) Å	b = 12.036(5) Å	b = 9.666(6) Å
	c = 6.502(3) Å	c = 6.6436(1) Å	c = 12.524(5) Å	c = 15.230(7) Å
Volume	1514.2(12) Å^3^	1497.61(5) Å^3^	1077.3(9) Å^3^	1082.16(9) Å^3^
Final R indices [I > 2σ(I)]	R1 = 0.0440 wR2 = 0.1193	R1 = 0.0579 wR2 = 0.1565	R1 = 0.0338 wR2 = 0.0848	R1 = 0.0316
CCDC No	2345576	2345579	2345573	JUGTAG [18]

## Data Availability

Crystallographic data for the structures presented in this paper have been deposited in the Cambridge Crystallographic Data Center as a Supplementary Publication (CCDC Nos 2345573–2345579). These data are provided free of charge by the Cambridge Crystallographic Data Centre Access Structures service www.ccdc.cam.ac.uk/structures/.

## Supplementary Materials

The following supporting information can be downloaded at: https://www.mdpi.com/article/10.3390/molecules29092133/s1, NMR spectra (^1^H 500 MHz, ^13^C NMR 126 MHz, ^31^P NMR 202 MHz), crystallographic data, and geometry of cation coordination spheres.
