# Supplementary material for: Cori Ester as the Ligand for Monovalent Cations
Source: Molecules. 2024 May 4;29(9):2133. doi: 10.3390/molecules29092133 (PMC11085912; doi:10.3390/molecules29092133)
Supplement: Supplementary file 1 [file molecules-29-02133-s001.zip › molecules-2975628-supplementary.pdf]

SUPPLEMENTARY MATERIALS

Cori ester as the ligand for monovalent cations

Krystyna Stępniak [1], Tadeusz Lis [2], Elżbieta Łastawiecka [1] and Anna E. Kozioł [1,*]

[1] Faculty of Chemistry, Maria Curie-Skłodowska University, 20-031 Lublin, Poland; krstepniak@poczta.onet.pl (KS); elzbieta.lastawiecka@mail.umcs.pl (EL)
[2] Faculty of Chemistry, University of Wrocław, 50-383 Wrocław, Poland; tadeusz.lis@uwr.edu.pl (TL – emeritus)
* Correspondence: anna.koziol@mail.umcs.pl (AEK)

1. NMR spectra
2. Crystalographic data
3. Cation coordination

NMR spectra of α-D-glucose 1-phosphate complexes

The NMR spectra were recorded using a Bruker Ascend (500 MHz) spectrometer in D_2O as the solvent, with a sample molarity of 0.05 mmol/mL at room temperature, unless specified otherwise. Chemical shifts (δ) are given in ppm relative to residual H_2O (^1H) as a reference. Coupling constants (*J*) are in Hz. The following abbreviations of signal patterns are as follows: s (singlet), d (doublet), t (triplet), q (quartet), m (multiplet), br (broad).

NMR data of α-D-Glucose 1-phosphate complexes

Dipotassium α-D-glucose 1-phosphate (Glc-1P2K**)**

^{1}H NMR (500 MHz, D_2O) δ ppm 3.28 (dd, *J* = 10.09, 9.14 Hz, 1 H, H_4), 3.37 (ddd, *J* = 9.62, 3.47, 1.73 Hz, 1 H, H_2), 3.63 (dd, *J* = 12.30, 5.04 Hz, 1 H, H_{6a}), 3.68 (t, *J* = 9.46 Hz, 1 H, H_3), 3.77 (dd, *J* = 12.14, 2.36 Hz, 1 H, H_{6b}), 3.82 (ddd, *J* = 10.17, 5.12, 2.36 Hz, 1 H, H_5), 5.34 (dd, *J* = 7.57, 3.47 Hz, 1 H, H_1);
^{13}C NMR (126 MHz, D_2O) δ ppm 60.71 (s), 69.76 (s), 71.91 (s), 72.25 (d, *J* = 6.36 Hz), 73.16 (s), 93.51 (d, *J* = 5.45 Hz);
^{31}P{^{1}H} NMR (202 MHz, D_2O) δ ppm 2.39;
^{31}P NMR (202 MHz, D_2O) δ ppm 2.37 (d, *J* = 7.46 Hz).

Disodium α-D-glucose 1-phosphate (Glc-1P2Na**)**

^{1}H NMR (500 MHz, D_2O) δ ppm 3.28 (dd, *J* = 10.25, 9.30 Hz, 1 H, H_4), 3.36 (ddd, *J* = 9.77, 3.47, 1.89 Hz, 1 H, H_2), 3.63 (dd, *J* = 12.30, 5.04 Hz, 1 H, H_{6a}), 3.68 (t, *J* = 9.46 Hz, 1 H, H_3), 3.77 (dd, *J* = 12.30, 2.52 Hz, 1 H, H_{6b}), 3.82 (ddd, *J* = 10.01, 5.28, 2.36 Hz, 1 H, H_5), 5.34 (dd, *J* = 7.57, 3.47 Hz, 1 H, H_1);
^{13}C NMR (126 MHz, D_2O) δ ppm 60.70 (s), 69.76 (s), 71.91 (s), 72.25 (d, *J* = 7.27 Hz), 73.16 (s), 93.51 (d, *J* = 5.45 Hz);
^{31}P{^{1}H} NMR (202 MHz, D_2O) δ ppm 2.35;
^{31}P NMR (202 MHz, D_2O) δ ppm 2.37 (d, J = 7.46 Hz).

Potassium sodium α-D-glucose 1-phosphate (Glc-1PKNa**)**

^{1}H NMR (500 MHz, D_2O) δ ppm 3.29 (t, *J* = 9.62 Hz, 1 H, H_4), 3.37 (ddd, *J* = 9.77, 3.47, 1.58 Hz, 1 H, H_2), 3.64 (dd, *J* = 12.30, 5.36 Hz, 1 H, H_{6a}), 3.68 (t, *J* = 9.50 Hz, 1 H, H_3), 3.77 (dd, *J* = 12.30, 2.60 Hz, 1 H, H_{6b}), 3.82 (ddd, *J* = 10.17, 5.12, 2.36 Hz, 1 H, H_6), 5.35 (dd, *J* = 7.57, 3.47 Hz, 1 H, H_1);
^{13}C NMR (126 MHz, D_2O) δ ppm 60.69 (s), 69.74 (s), 71.91 (s), 72.23 (d, *J* = 6.36 Hz), 73.14 (s), 93.53 (d, *J* = 4.54 Hz);
^{31}P{^{1}H} NMR (202 MHz, D_2O) δ ppm 2.33;
^{31}P NMR (202 MHz, D_2O) δ ppm 2.32 (d, *J* = 4.98 Hz).

Table S1. Chemical shift and coupling constant for selected atoms.

	**C1**		**H6a**		**H6b**	
	δ [ppm]	*J* [Hz]	δ [ppm]	*J* [Hz]	δ [ppm]	*J* [Hz]
Glc-1P2K	93.51	5.45	3.63	12.30; 5.04	3.77	12.14; 2.36
Glc-1P2Na	93.51	5.45	3.63	12.30; 5.04	3.77	12.14; 2.52
Glc-1PKNa	93.51	4.54	3.64	12.30; 5.36	3.77	12.14; 2.60

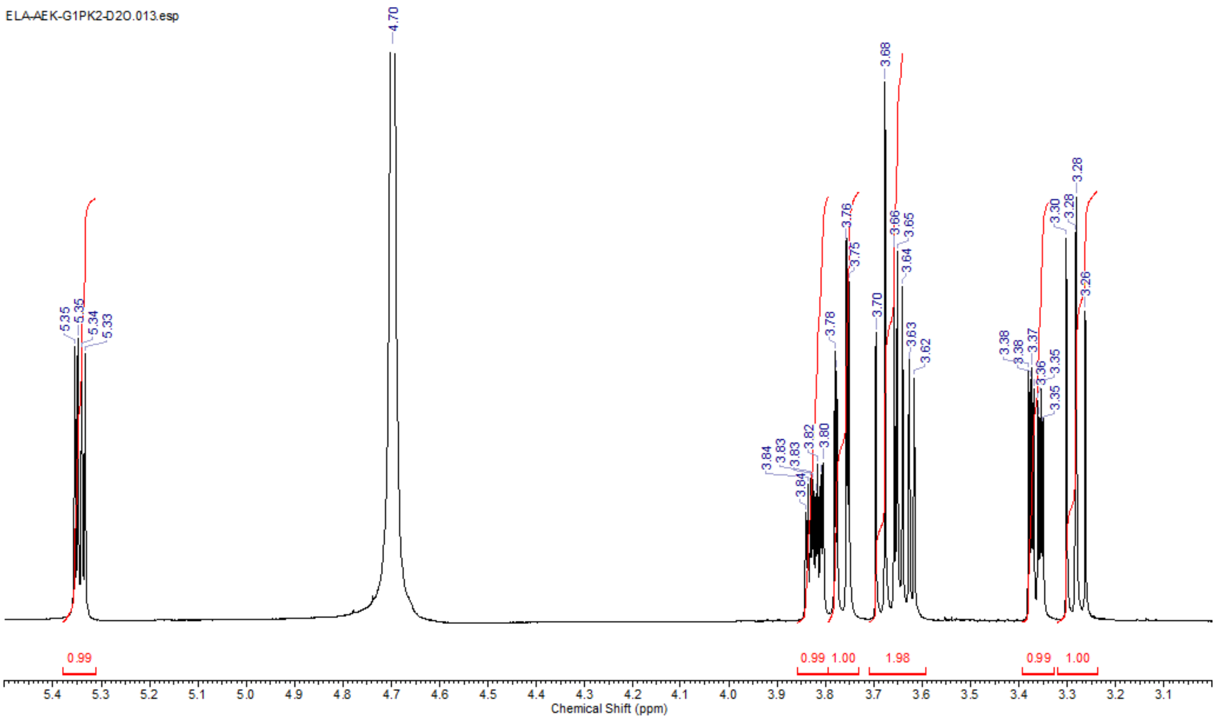

Figure S1. ^{1}H NMR (D_2O, 500 MHz) spectra of dipotassium α-D-glucose 1-phosphate (Glc-1P2K).

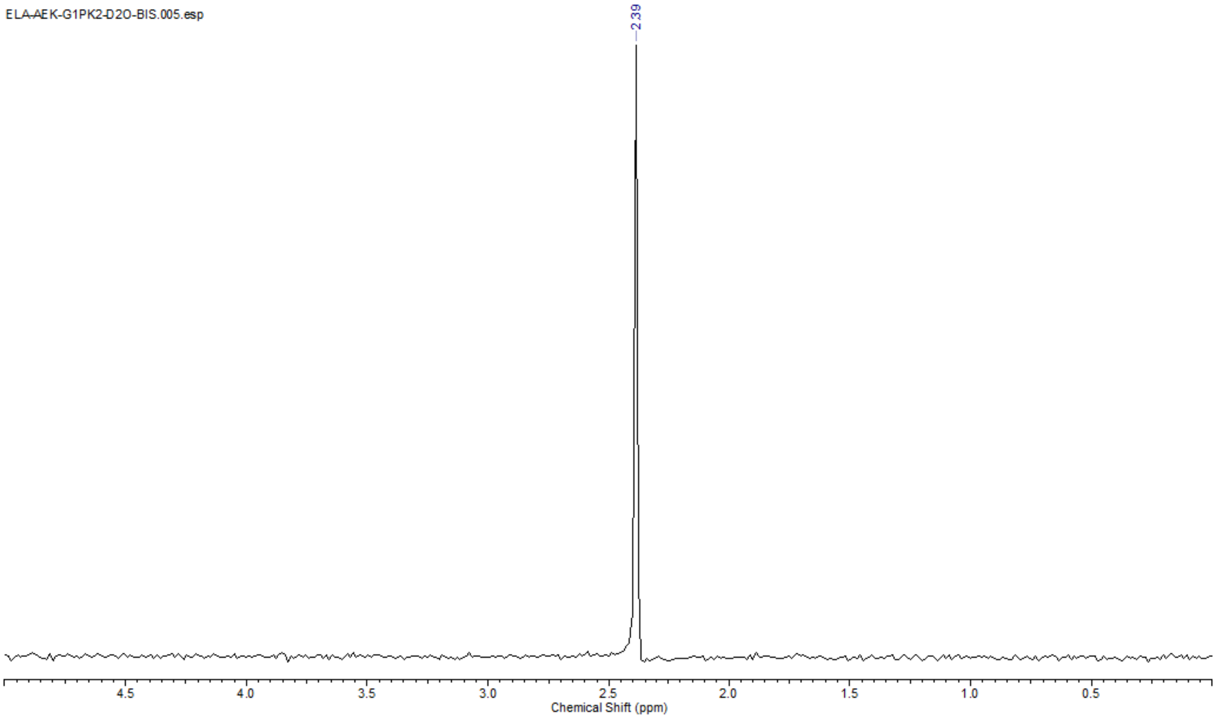

Figure S2. ^{31}P NMR of dipotassium α-D-glucose 1-phosphate (Glc-1P2K) in D_2O, with ^{1}H decoupling, at 202 MHz.

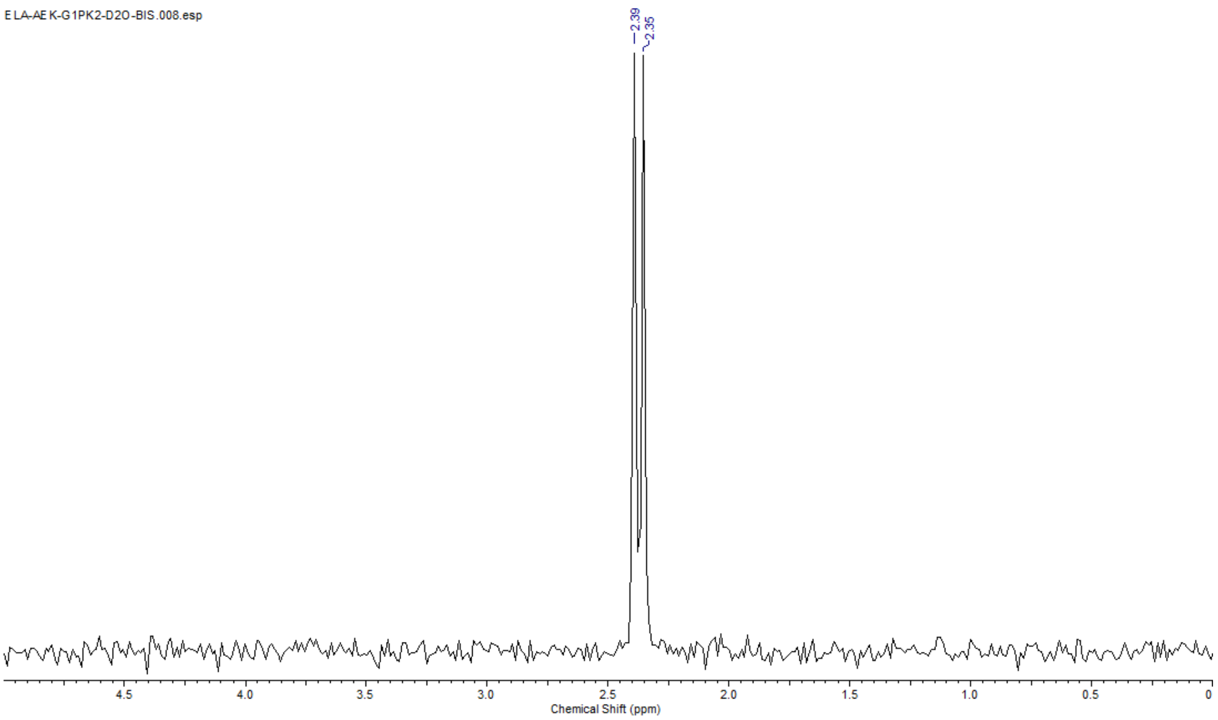

Figure S3. ^{31}P NMR of dipotassium α-D-glucose 1-phosphate (Glc-1P2K) in D_2O, with no decoupling, at 202 MHz.

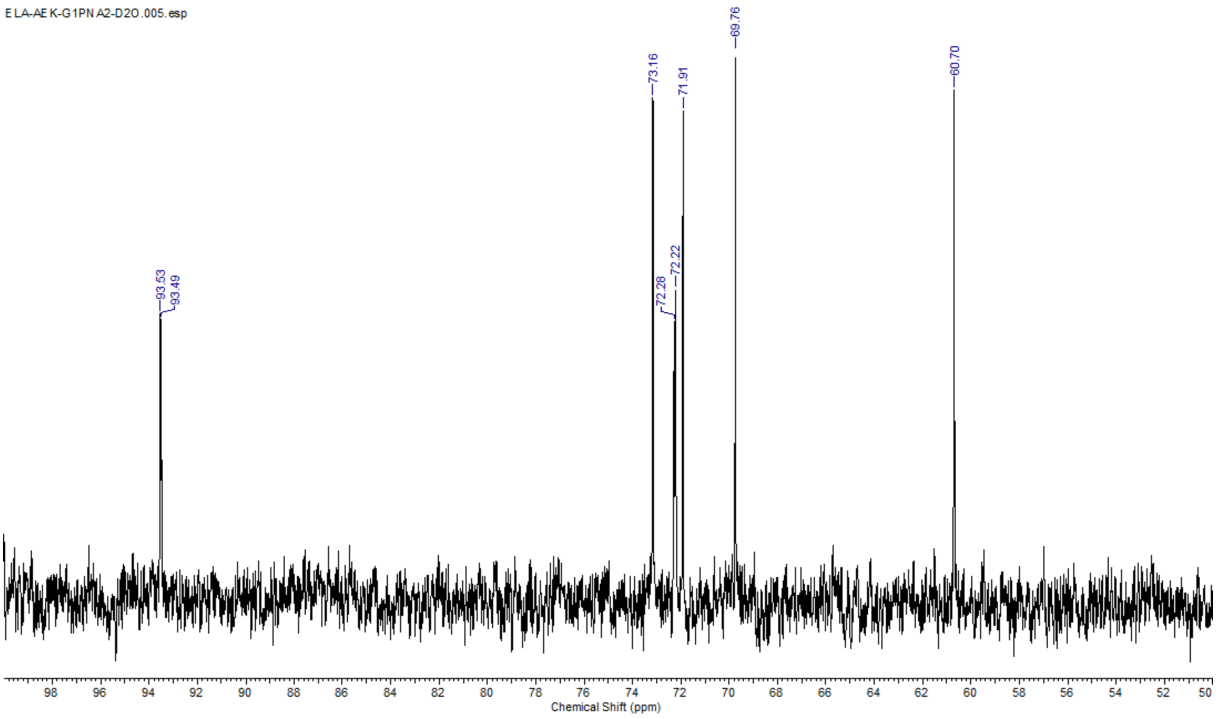

Figure S4. ^{13}C NMR (D_2O, 126 MHz) spectra of dipotassium α-D-glucose 1-phosphate (Glc-1P2K)

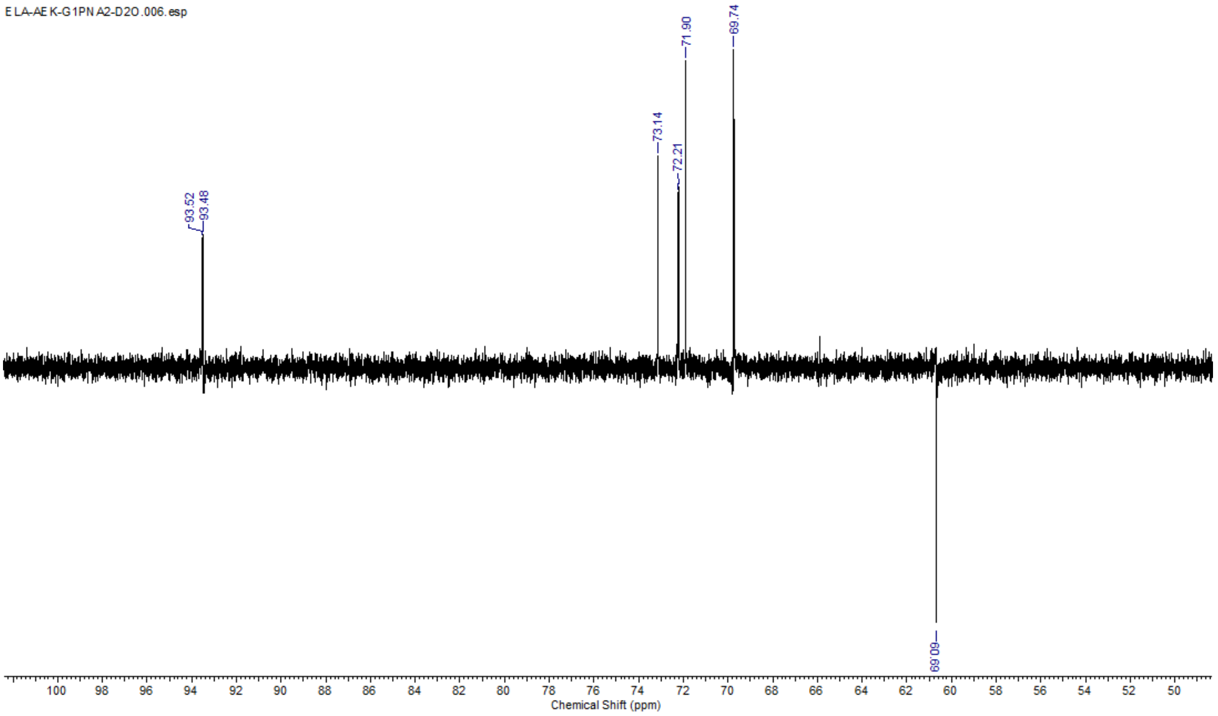

Figure S5. DEPT 135 spectra of dipotassium α-D-glucose 1-phosphate (Glc-1PK$_2$ Glc-1P2K) (D_2O, 126 MHz).

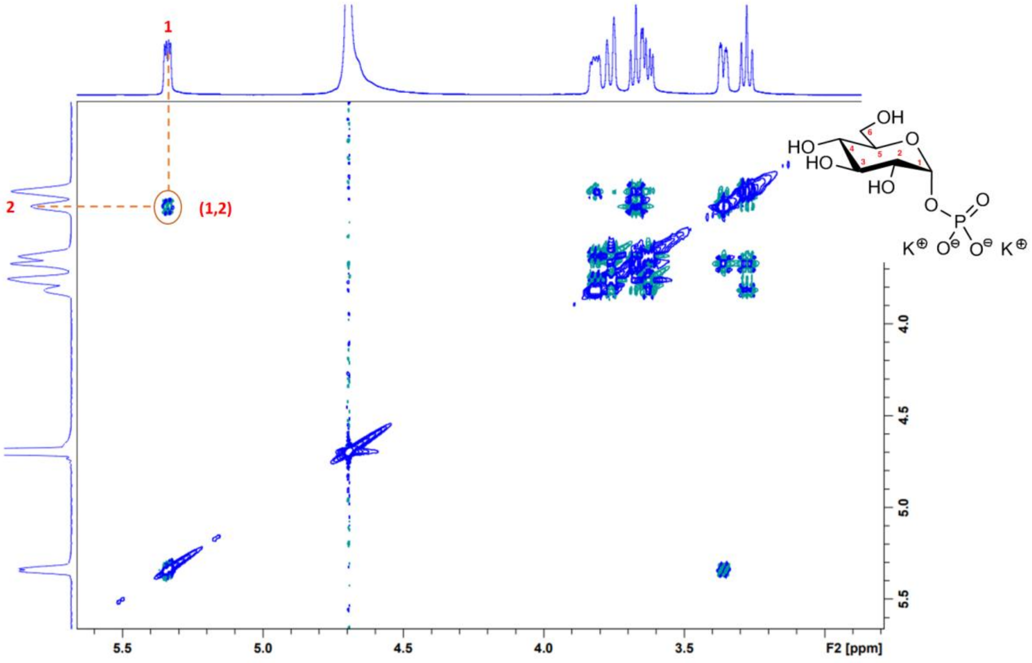

Figure S6. 500 MHz ^{1}H 2D NOESY spectrum of dipotassium α-D-glucose 1-phosphate (Glc-1P2K).

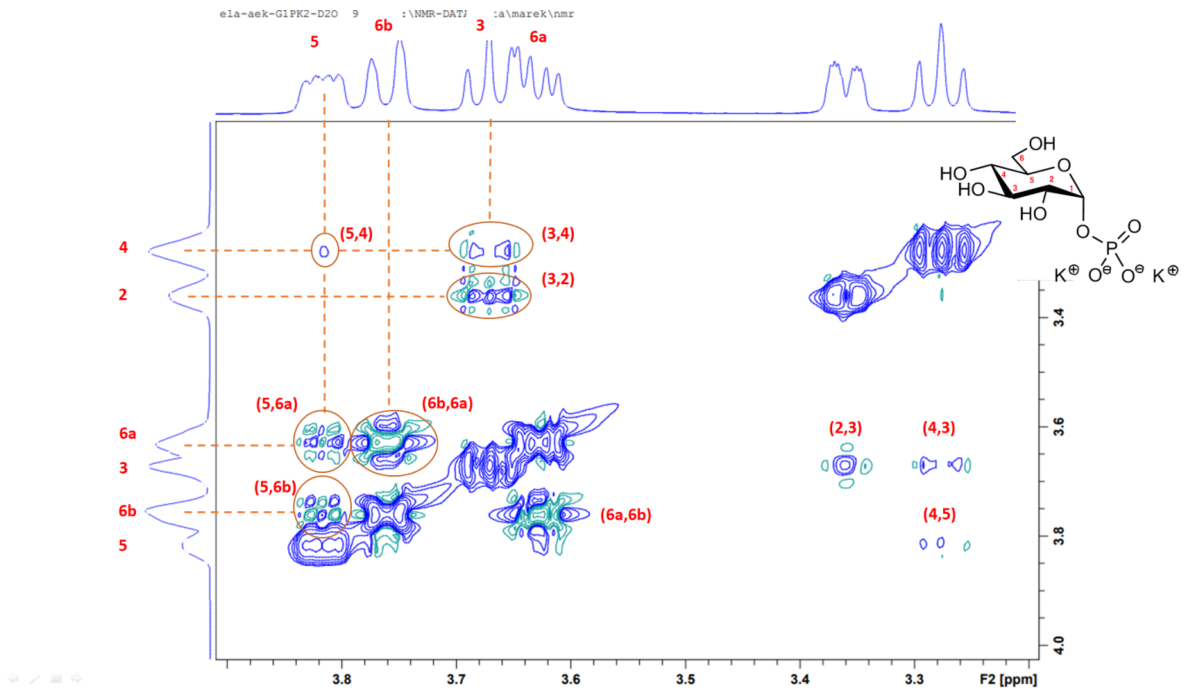

Figure S7. Excerpts of the 500 MHz ^{1}H 2D NOESY spectrum of dipotassium α-D-glucose 1-phosphate (Glc-1P2K).

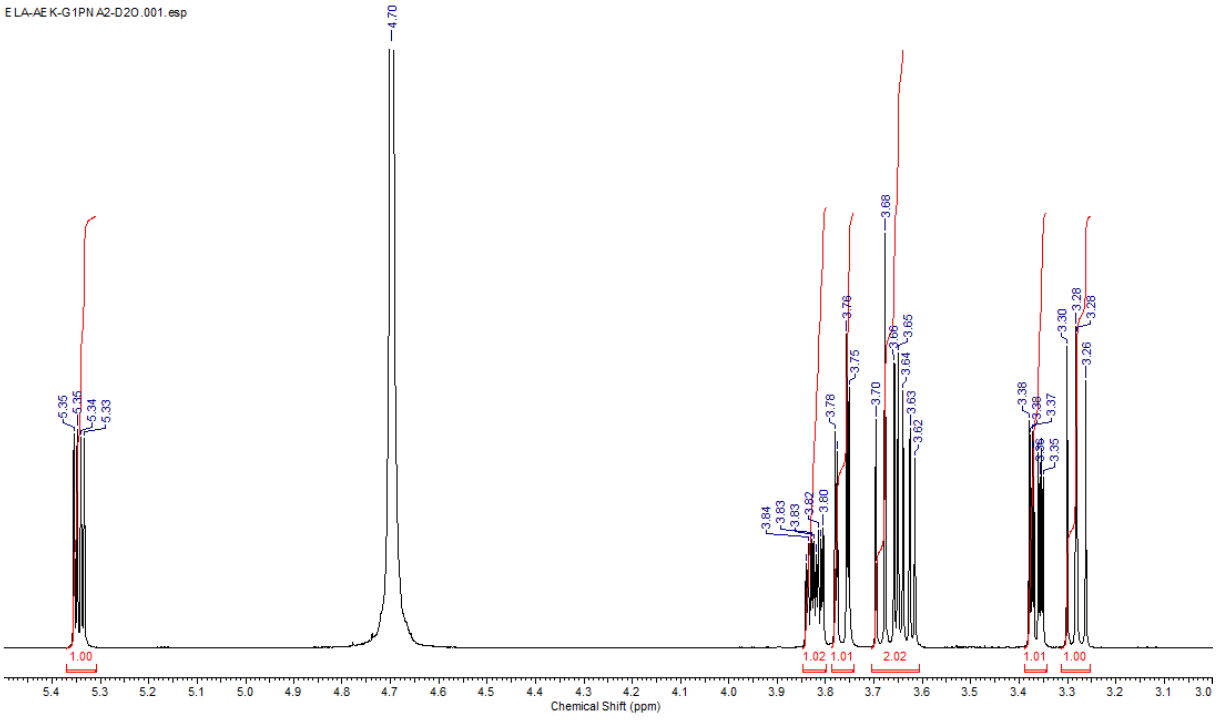

Figure S8. ^{1}H NMR spectra of disodium α-D-glucose 1-phosphate (Glc-1P2Na) (D_2O, 500 MHz)

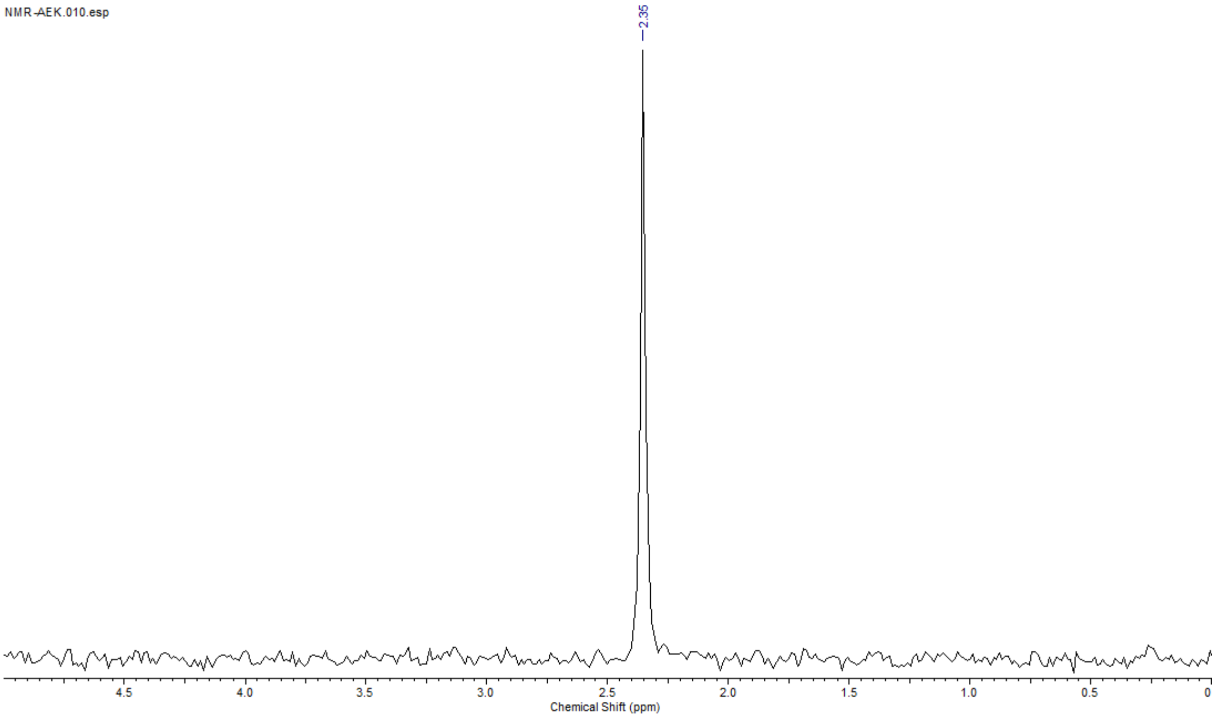

Figure S9. ^{31}P NMR of disodium α-D-glucose 1-phosphate (Glc-1P2Na) in D_2O, with 1H decoupling, at 202 MHz.

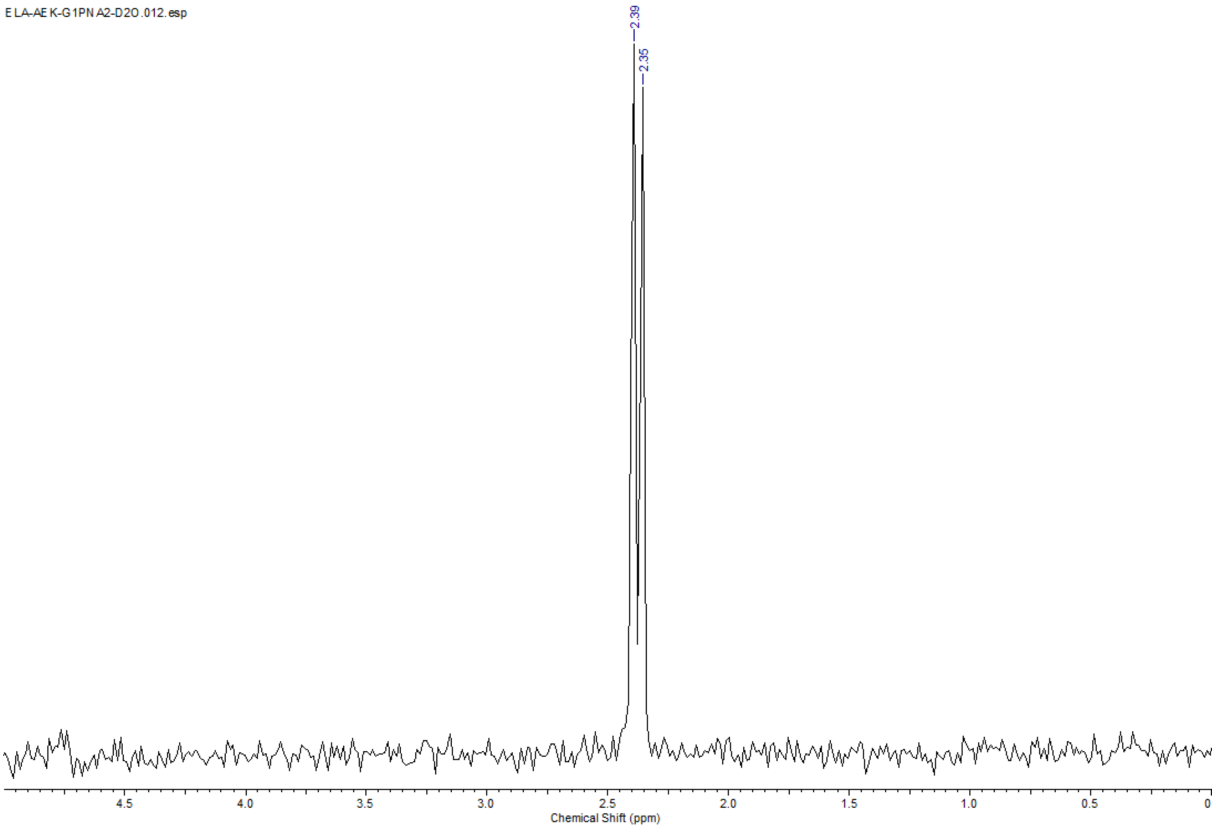

Figure S10. ^{31}P NMR of disodium α-D-glucose 1-phosphate (Glc-1P2Na) in D_2O, with no decoupling, at 202 MHz.

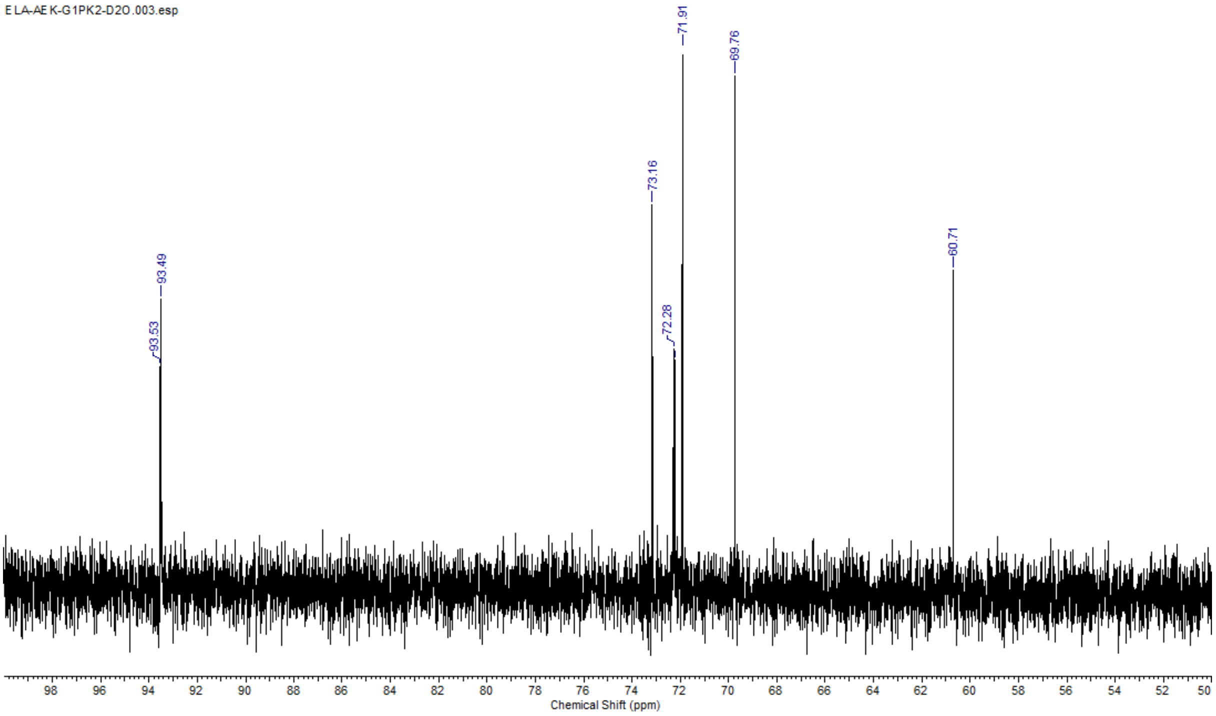

Figure S11. ^{13}C NMR spectra of disodium α-D-glucose 1-phosphate (Glc-1P2Na) (D_2O, 126 MHz)

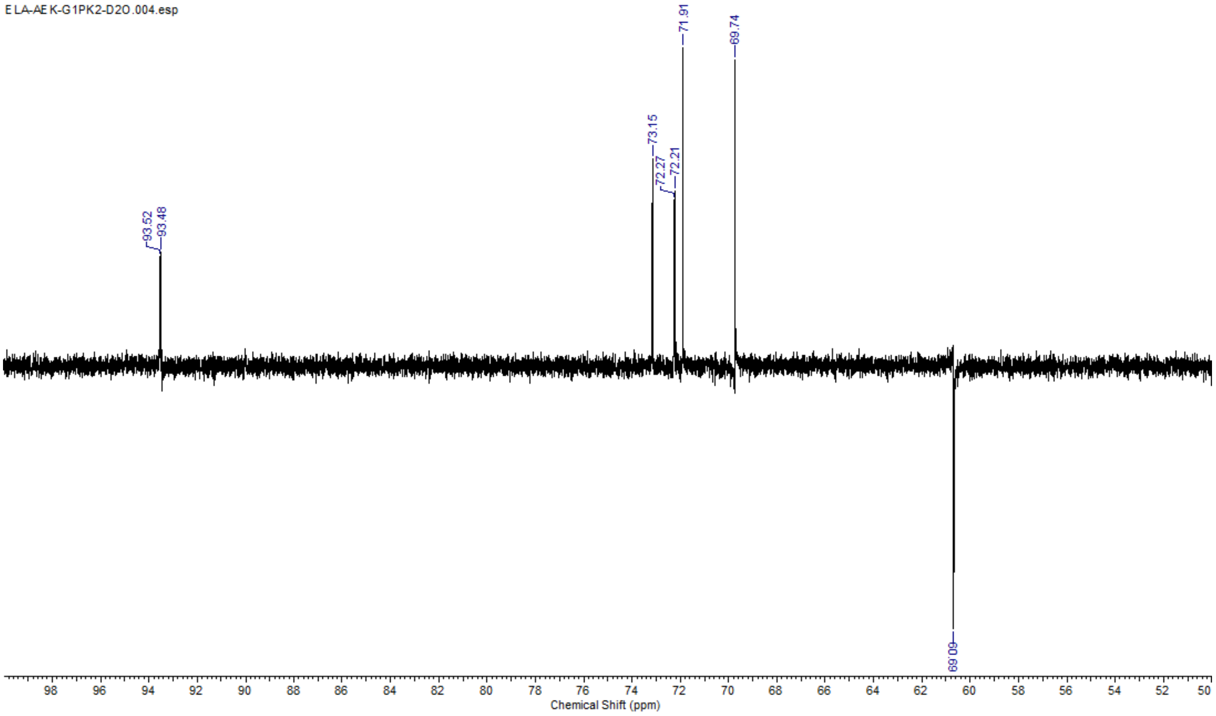

Figure S12. DEPT 135 spectra of disodium α-D-glucose 1-phosphate (Glc-1P2Na) (D_2O, 126 MHz).

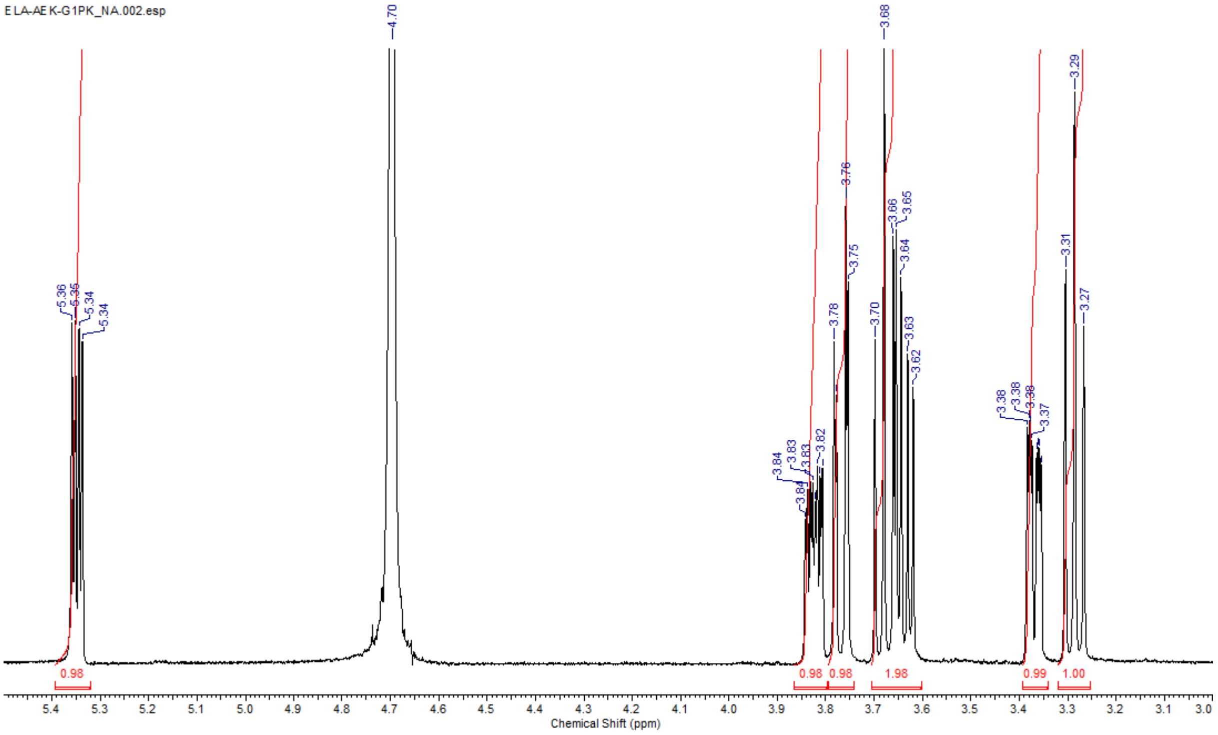

Figure S13. ^{1}H NMR spectra of potassium sodium α-D-glucose 1-phosphate α-D-Glucose (Glc-1PKNa) (D_2O, 500 MHz)

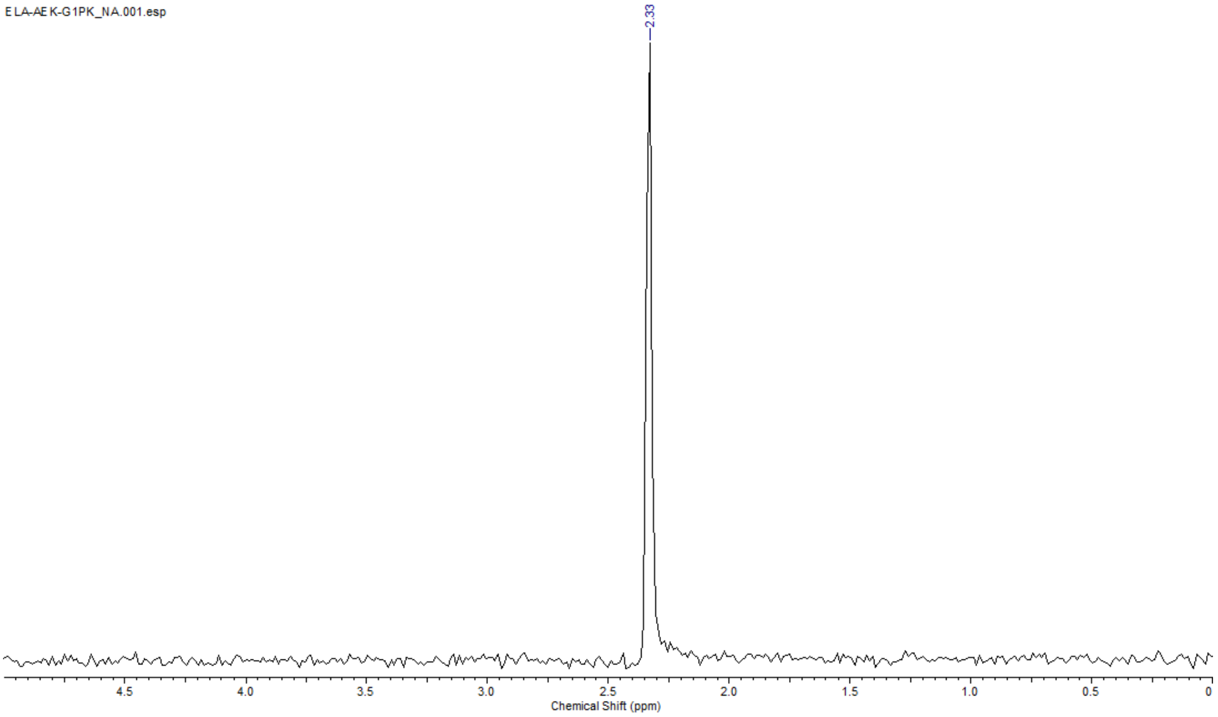

Figure S14. ^{31}P NMR of potassium sodium α-D-glucose 1-phosphate (Glc-1PKNa) in D_2O, with 1H decoupling, at 202 MHz.

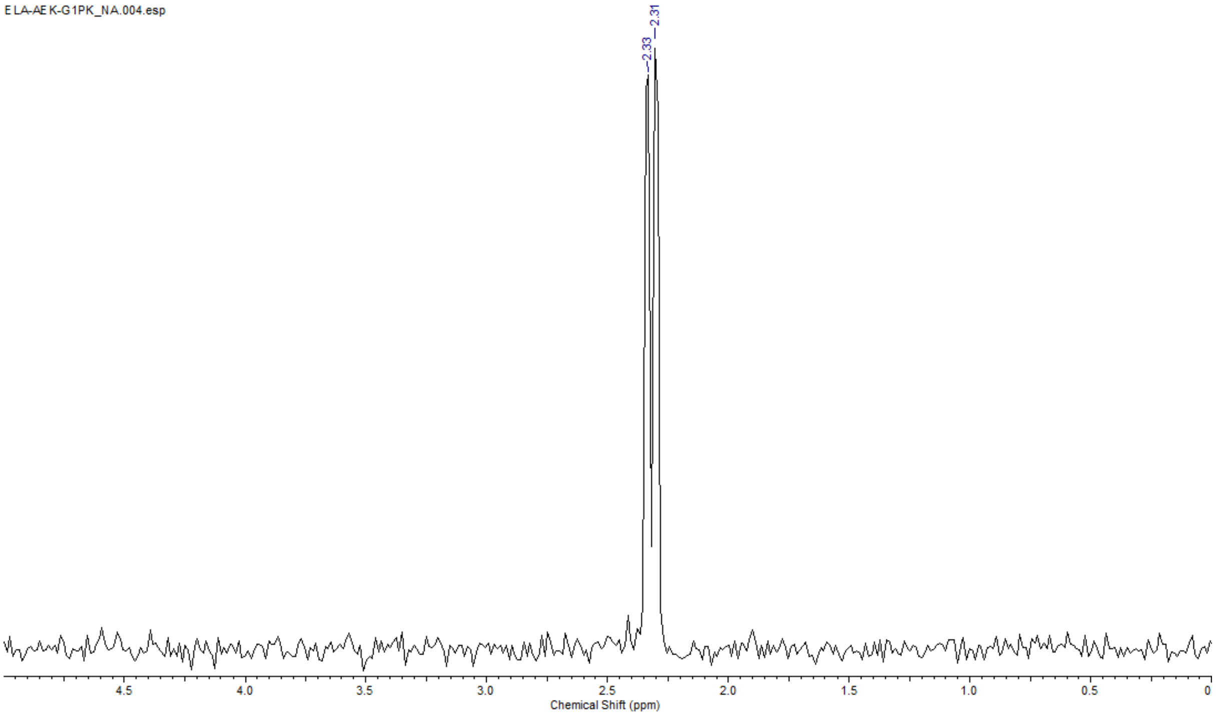

Figure S15. ^{31}P NMR of potassium sodium α-D-glucose 1-phosphate (Glc-1PKNa) in D_2O, with no decoupling, at 202 MHz.

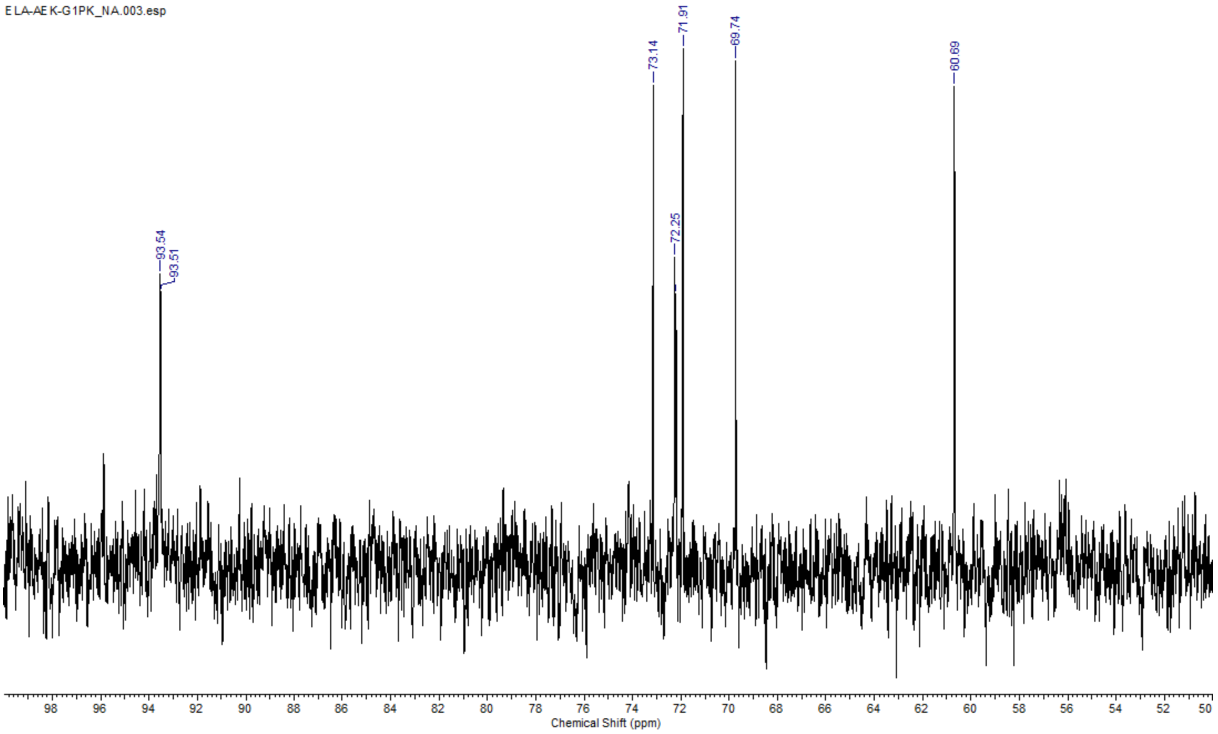

Figure S16. ^{13}C NMR spectra of potassium sodium α-D-glucose 1-phosphate (Glc-1PKNa) (D_2O, 126 MHz)

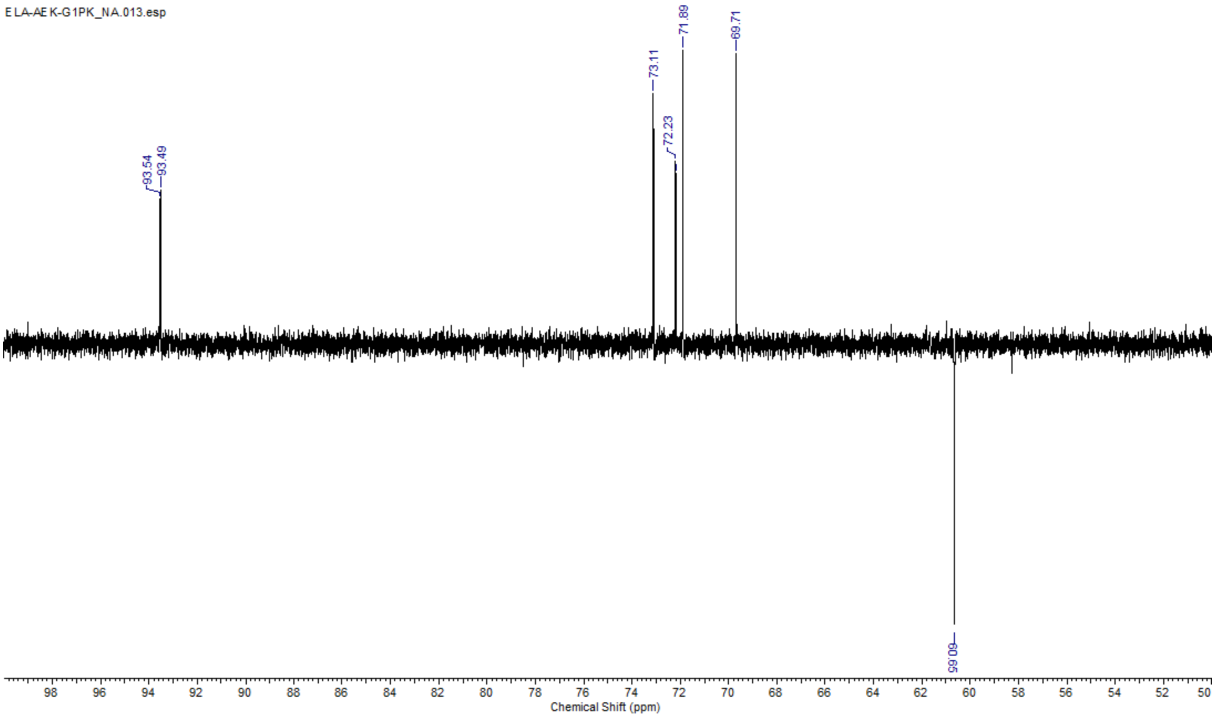

Figure S17. DEPT 135 spectra of potassium sodium α-D-glucose 1-phosphate (Glc-1PKNa) (D_2O, 126 MHz).

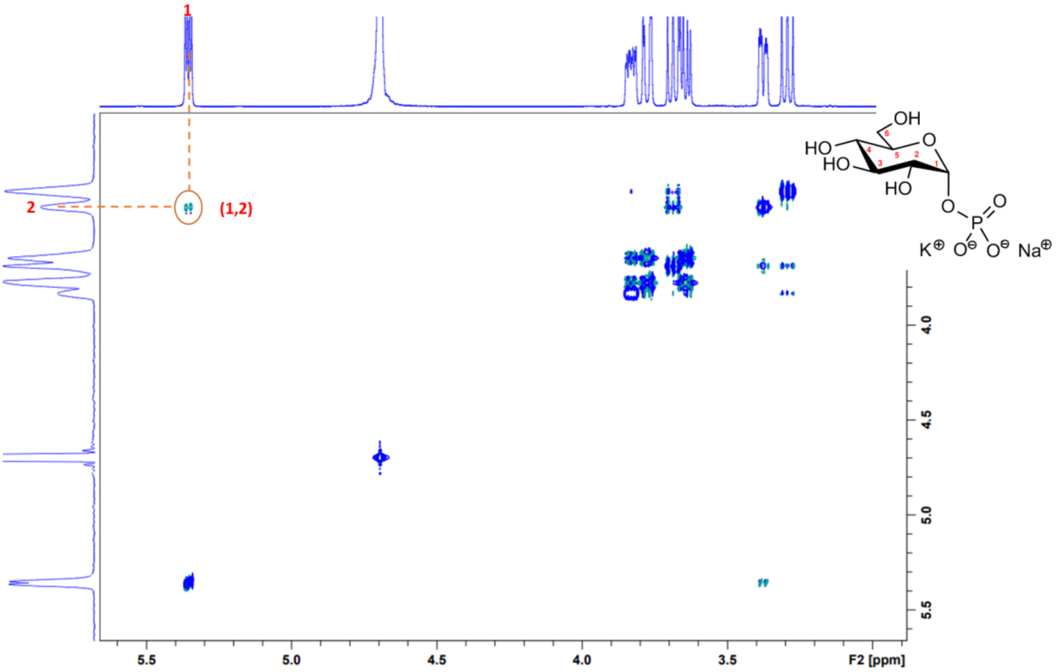

Figure S18. 500 MHz ^{1}H 2D NOESY spectrum of potassium sodium α-D-glucose 1-phosphate (Glc-1PKNa)

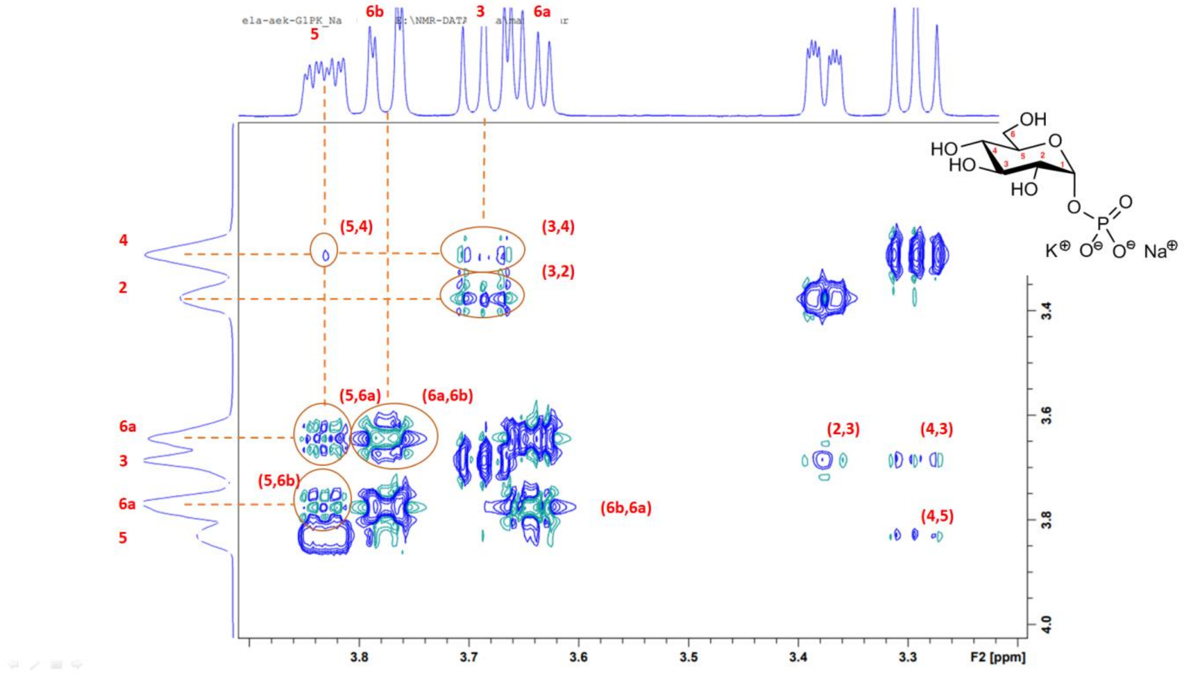

Figure S19. Excerpts of the 500 MHz ^{1}H 2D NOESY spectrum of potassium sodium α-D-glucose 1-phosphate (Glc-1PKNa)

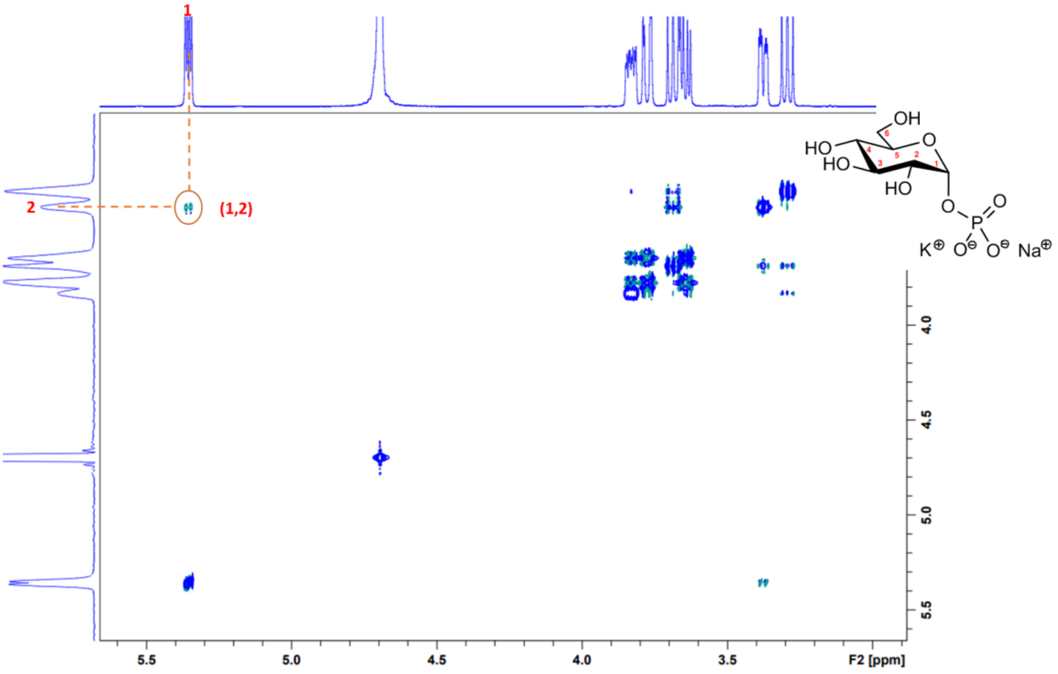

Figure S20. 500 MHz ^{1}H 2D NOESY spectrum of potassium sodium α-D-glucose 1-phosphate (Glc-1PKNa)

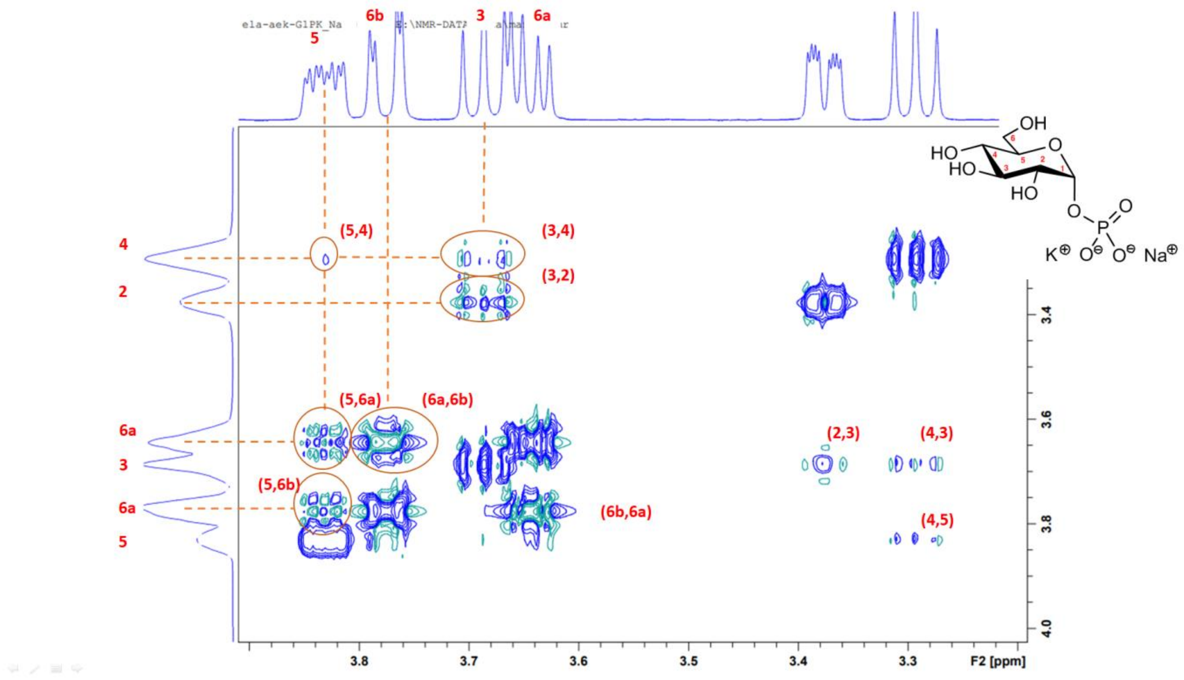

Figure S21. Excerpts of the 500 MHz ^{1}H 2D NOESY spectrum of potassium sodium α-D-glucose 1-phosphate (Glc-1PKNa)

CRYSTALLOGRAPHIC DATA

Table S2. Potassium sodium α-D-glucose 1-phosphate tetrahydrate

**Identification code**	**G1P-KNa**
**CCDC No**	**2345577**
Formula	C6 H19 K1.50 Na0.50 O13 P
Formula weight	400.33
Wavelength	1.54184 Å
Crystal system	Orthorhombic
Space group	$P\ 2_12_12$
Unit cell dimensions	a = 16.8360(4) Å
	b = 13.4089(3) Å
	c = 6.6729(1) Å
Volume	1506.42(5) $Å^3$
Z	4
Density (calculated)	1.765 g/cm^3
Absorption coefficient	6.121 mm^{-1}
F(000)	832
Theta range for data collection	4.215 to 76.361°.
Index ranges	-21<=h<=19, -16<=k<=16, -8<=l<=6
Reflections collected	10925
Independent reflections	3123 [R(int) = 0.0314]
Completeness to theta = 67.684°	99.9 %
Data / restraints / parameters	3123 / 0 / 213
Goodness-of-fit on F^2	1.062
Final R indices [I>2sigma(I)]	R1 = 0.0321, wR2 = 0.0852
R indices (all data)	R1 = 0.0343, wR2 = 0.0873
Absolute structure parameter	-0.004(6)
Largest diff. peak and hole	0.46 and -0.45 $e.Å^{-3}$

Table S3. Disodium α-D-glucose 1-phosphate pentahydrate

**Identification code**	**Glc-1P2Na-5H2O**
**CCDC No**	**2345578**
Empirical formula	C6 H21 Na2 O14 P
Formula weight	394.18
Wavelength	1.54184 Å
Crystal system	Orthorhombic
Space group	$P\ 2_12_12_1$
Unit cell dimensions	a = 6.6347(5) Å
	b = 8.7585(9) Å
β= 99.147(2)°.	c = 27.129(2) Å
Volume	1576.5(2) Å^3
Z	4
Density (calculated)	1.661 g/cm^3
Absorption coefficient	2.783 mm^{-1}
F(000)	824
Theta range for data collection	5.307 to 76.416°
Index ranges	-8<=h<=8, -10<=k<=10, -33<=l<=24
Reflections collected	10188
Independent reflections	3208 [R(int) = 0.0381]
Completeness to theta = 67.684°	99.7 %
Data / restraints / parameters	3208 / 0 / 253
Goodness-of-fit on F^2	1.161
Final R indices [I>2sigma(I)]	R1 = 0.0277, wR2 = 0.0613
R indices (all data)	R1 = 0.0367, wR2 = 0.0814
Absolute structure parameter	0.035(16)
Extinction coefficient	0.0016(3)
Largest diff. peak and hole	0.27 and -0.26 e.Å^{-3}

Table S4. Disodium α-D-glucose 1-phosphate trihemihydrate (redetermination – CIMDUX01)

**Identification code**	**G1P2NA-3.5H2O**
**CCDC No**	**23455775**
Empirical formula	C6 H18 Na2 O12.50 P
Formula weight	367.15
Wavelength	1.54184 Å
Crystal system	Monoclinic
Space group	*C* 2
Unit cell dimensions	a = 8.4325(2) Å
	b = 10.1983(2) Å
	c = 16.5857(4) Å
	β= 99.147(2)°
Volume	1408.19(6) $Å^3$
Z	4
Density (calculated)	1.732 g/cm^3
Absorption coefficient	2.986 mm^{-1}
F(000)	764
Theta range for data collection	5.403 to 76.054°.
Index ranges	-10<=h<=10, -12<=k<=12, -19<=l<=20
Reflections collected	2612
Independent reflections	2017 [R(int) = 0.0242]
Completeness to theta = 67.684°	99.6 %
Data / restraints / parameters	2017 / 1 / 265
Goodness-of-fit on F^2	1.043
Final R indices [I>2sigma(I)]	R1 = 0.0243, wR2 = 0.0635
R indices (all data)	R1 = 0.0245, wR2 = 0.0639
Absolute structure parameter	-0.06(2)
Extinction coefficient	0.0081(4)
Largest diff. peak and hole	0.27 and -0.23 e.$Å^{-3}$

Table S5. Dipotassium α-D-glucose 1-phosphate dihydrate (redetermination – KGLUCP02)

**Identification code**	**G1P2K2H2O**
**CCDC No**	**2345574**
Empirical formula	C6 H15 K2 O11 P
Formula weight	372.35
Wavelength	1.54184 Å
Crystal system	Monoclinic
Space group	$P\,2_1$
Unit cell dimensions	a = 7.5234(3) Å
β= 110.576(5)°	b = 9.0643(3) Å
	c = 10.4588(4) Å
Volume	667.73(5) $Å^3$
Z	2
Density (calculated)	1.852 g/cm^3
Absorption coefficient	7.959 mm^{-1}
F(000)	384
Theta range for data collection	4.516 to 76.141°.
Index ranges	-9<=h<=8, -8<=k<=11, -11<=l<=13
Reflections collected	4319
Independent reflections	2178 [R(int) = 0.0272]
Completeness to theta = 67.684°	99.8 %
Data / restraints / parameters	2178 / 1 / 217
Goodness-of-fit on F^2	1.075
Final R indices [I>2sigma(I)]	R1 = 0.0294, wR2 = 0.0774
R indices (all data)	R1 = 0.0298, wR2 = 0.0779
Absolute structure parameter	0.063(10)
Largest diff. peak and hole	0.25 and -0.50 e.$Å^{-3}$

Table S6. Diammonium α-D-glucose 1-phosphate trihydrate

**Identification code**	**G1P2NH4**
**CCDC No**	**2345576**
Empirical formula	C6 H25 N2 O12 P
Formula weight	348.25
Wavelength	0.71069 Å
Crystal system	Orthorhombic
Space group	$P\ 2_12_12$
Unit cell dimensions	a = 13.756(7) Å
	b = 16.929(7) Å
	c = 6.502(3) Å
Volume	1514.2(12) $Å^3$
Z	4
Density (calculated)	1.528 g/cm^3
Absorption coefficient	0.244 mm^{-1}
F(000)	744
Theta range for data collection	2.825 to 32.568°
Index ranges	-20<=h<=20, -22<=k<=25, -9<=l<=9
Reflections collected	3297
Independent reflections	3297 [R(int) = 0.028]
Completeness to theta = 25.240°	84.8 %
Data / restraints / parameters	3297 / 0 / 204
Goodness-of-fit on F^2	1.083
Final R indices [I>2sigma(I)]	R1 = 0.0440, wR2 = 0.1193
R indices (all data)	R1 = 0.0440, wR2 = 0.1193
Absolute structure parameter	-0.02(8)
Largest diff. peak and hole	0.57 and -0.35 e. $Å^{-3}$

Table S7. Ammonium sodium α-D-glucose 1-phosphate tetrahydrate

**Identification code**	**Glc-1P(NH4)Na**
**CCDC No**	**2345579**
Empirical formula	C6 H25 N1.50 Na0.50 O13 P
Formula weight	368.74
Wavelength	1.54184 Å
Crystal system	Orthorhombic
Space group	$P\ 2_12_12$
Unit cell dimensions	a = 16.9272(3) Å
	b = 13.3171(3) Å
	c = 6.6436(1) Å
Volume	1497.61(5) $Å^3$
Z	4
Density (calculated)	1.635 g/cm^3
Absorption coefficient	2.462 mm^{-1}
F(000)	784
Theta range for data collection	5.226 to 76.184°
Index ranges	-21<=h<=18, -16<=k<=16, -8<=l<=7
Reflections collected	10118
Independent reflections	3069 [R(int) = 0.0359]
Completeness to theta = 67.684°	99.7 %
Data / restraints / parameters	3069 / 0 / 200
Goodness-of-fit on F^2	1.066
Final R indices [I>2sigma(I)]	R1 = 0.0579, wR2 = 0.1565
R indices (all data)	R1 = 0.0647, wR2 = 0.1609
Absolute structure parameter	0.008(14)
Largest diff. peak and hole	0.64 and -0.62 $e.Å^{-3}$

Table S8. Ammonium potassium α-D-glucose 1-hydrogenphosphate

**Identification code**	**Glc-1PH(NH4)K**
**CCDC No**	**2345573**
Empirical formula	C6 H14.75 K0.31 N0.69 O9 P
Formula weight	283.74
Wavelength	1.5418 Å
Crystal system	Orthorhombic
Space group	$P\ 2_12_12_1$
Unit cell dimensions	a = 7.147(4) Å
	b = 12.036(5) Å
	c = 12.524(5) Å
Volume	1077.3(9) $Å^3$
Z	4
Density (calculated)	1.749 g/cm^3
Absorption coefficient	3.793 mm^{-1}
F(000)	594
Theta range for data collection	5.096 to 74.984°.
Index ranges	-8<=h<=0, -15<=k<=14, -15<=l<=15
Reflections collected	4183
Independent reflections	2211 [R(int) = 0.0427]
Completeness to theta = 67.680°	99.9 %
Data / restraints / parameters	2211 / 0 / 164
Goodness-of-fit on F^2	1.048
Final R indices [I>2sigma(I)]	R1 = 0.0338, wR2 = 0.0851
R indices (all data)	R1 = 0.0382, wR2 = 0.0873
Absolute structure parameter	0.033(12)
Largest diff. peak and hole	0.56 and -0.42 e.$Å^{-3}$

Table S9. Potassium α-D-glucose 1-hydrogenphosphate [18]

**Identification code**	**Glc-1PHK (JUGTAG)**
Formula	C6 H12 O9 P K
Formula weight	298.23
Crystal system	Orthorhombic
Space group	$P\ 2_12_12_1$
Unit cell dimensions	a = 7.35(4) Å
	b = 9.666(6) Å
	c = 15.230(7) Å
Volume	1082.16(9) Å^3
Density	1.83
R1	R1=0.0316

Potassium coordination polyhedron for JUGTAG – Glc-1PHK

LK = 6 TRIGONAL PRISM

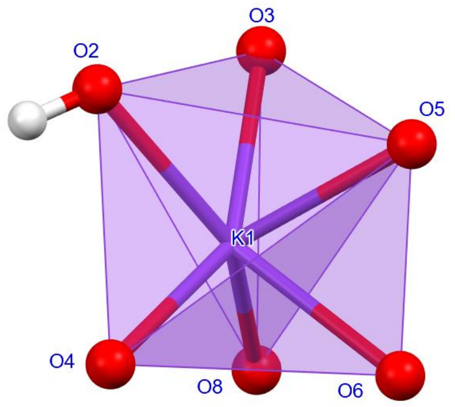

CATION COORDINATION

CSD search for complexes of chemical composition C+H+O+cation (Na/K); R1 < 10%

Cation oordination number	Na CN = 5	Na CN = 6	K CN = 6	K CN = 8
Ionic radii (Å) [Shannon]	1.14	1.16	1.52	1.65
Na/K….O distance range (Å)	2.13 – 2.62	2.15 – 2.74	2.51 – 3.27	2.59 – 3.17
No of spheres	*18*	*48*	*7*	*35*
No of bonds	*18x5*	*48x6*	*7x6*	*35x8*

Table S10. Cation coordination in the Glc-1P2K 2H_2O crystal

<table>
<tr>
<td>LK = 6
K(1)-O(6) 2.716(3)
K(1)-O(11) 2.712(3)
K(1)-O(4)#1 2.717(3)
K(1)-O(5)#2 2.736(2)
K(1)-O(3)#3 2.760(3)
K(1)-O(1W) 2.881(3)

#1 x-1,y,z
#2 -x,y-1/2,-z+1
#3 -x+1,y-1/2,-z+1</td>
<td>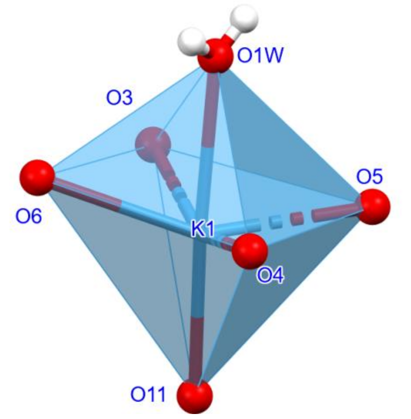

OCTAHEDRON (OC-6)</td>
</tr>
<tr>
<td>LK = 8
K(2)-O(2) 2.831(3)
K(2)-O(2)#8 3.085(3)
K(2)-O(3)#8 3.072(3)
K(2)-O(6)#5 2.962(3)
K(2)-O(12)#7 3.013(3)
K(2)-O(1W)#5 2.939(3)
K(2)-O(2W) 2.797(4)
K(2)-O(2W)#6 2.943(4)

#5 x,y,z-1 #6 -x+1,y+1/2,-z
#7 x+1,y,z #8 -x+1,y-1/2,-z</td>
<td>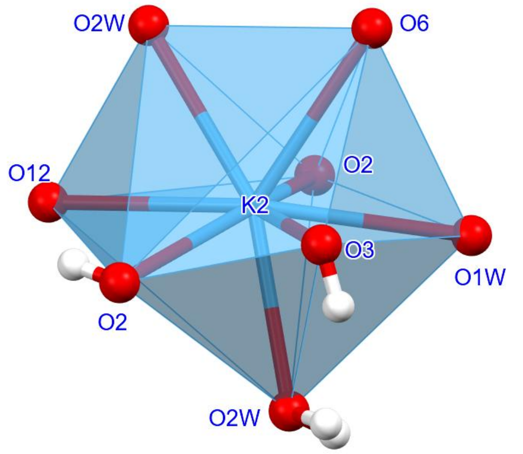

DODECAHEDRON (DD-8)</td>
</tr>
</table>

Table S11. Cation coordination in the Glc-1P2Na 3.5 H_2O crystal

**LK = 5**<br>Na(1)-O(13) 2.314 (2)<br>Na(1)-O(2)#1 2.424(2)<br>Na(1)-O(1W) 2.360(2)<br>Na(1)-O(2W) 2.347(2)<br>Na(1)-O(3W) 2.431(2)	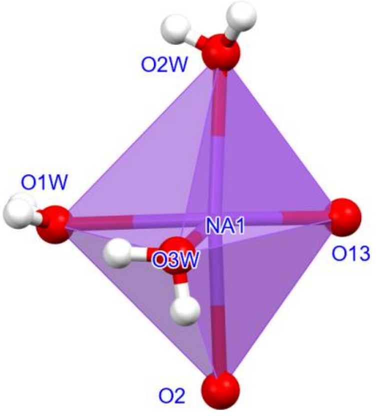<br>TRIGONAL BIPYRAMID (TBPY-5)
**LK =6** (2-fold axis)<br>Na(2)-O(5) 2.440(2)<br>Na(2)-O(6) 2.394(2)<br>Na(2)-O(5)#2 2.440(2)<br>Na(2)-O(6)#2 2.394(2)<br>Na(2)-O(4)#3 2.405(2)<br>Na(2)-O(4)#4 2.405(2)	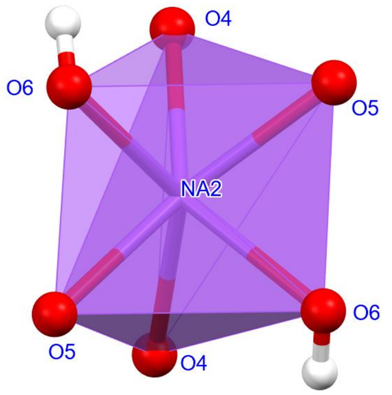<br>TRIGONAL PRISM (TPR-6)
**LK =6** (2-fold axis)<br>Na(3)-O(13) 2.526(2)<br>Na(3)-O(13)#5 2.526(2)<br>Na(3)-O(2W) 2.397(2)<br>Na(3)-O(2W)#5 2.397(2)<br>Na(3)-O(3W)#6 2.417(2)<br>Na(3)-O(3W)#7 2.417(2)	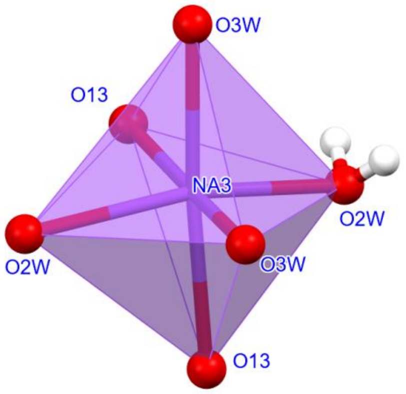<br>OCTAHEDRON (OC-6)

Symmetry transformations used to generate equivalent atoms:
#1 x-1/2,y-1/2,z #2 -x+1,y,-z #3 -x+1/2,y-1/2,-z
#4 x+1/2,y-1/2,z #5 -x+1,y,-z+1 #6 -x+1/2,y+1/2,-z+1
#7 x+1/2,y+1/2,z #8 x-1/2,y+1/2,z

Table S12. Cation coordination in the Glc-1P2Na 5 H_2O crystal

<table>
<tr>
<td>LK = 5

Na(1)-O(5) 2.471(3)
Na(1)-O(6) 2.317(3)
Na(1)-O(1W) 2.405(4)
Na(1)-O(2W) 2.356(4)
Na(1)-O(3W) 2.331(4)</td>
<td>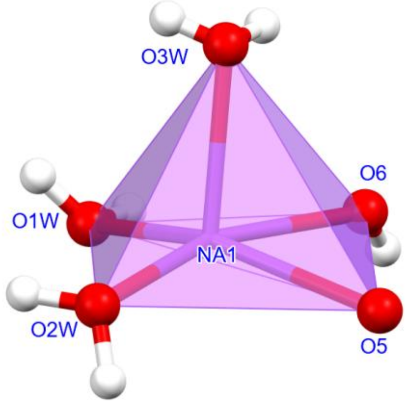

TETRAGONAL PYRAMID (SPY-5)</td>
</tr>
<tr>
<td>LK = 6

Na(2)-O(1)#2 2.525(3)
Na(2)-O(2)#2 2.492(3)
Na(2)-O(13) 2.344(3)
Na(2)-O(13)#2 2.610(3)
Na(2)-O(4W) 2.416(3)
Na(2)-O(4W)#1 2.397(4)</td>
<td>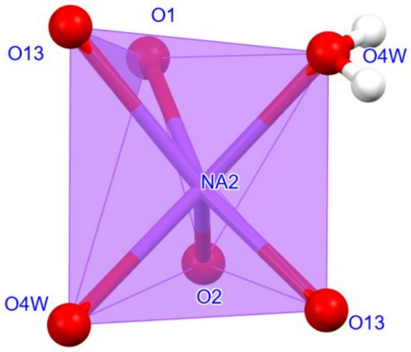

TRIGONAL PRISM (TPR-6)</td>
</tr>
</table>

#1 x+1/2,-y+3/2,-z
#2 x-1/2,-y+3/2,-z

Table S13. Cation coordination in the Glc-1PKNa crystal

<table>
<tr><td>LK =6 (2-fold axis)
K(1)-O(2) 2.980(3)
K(1)-O(3) 2.735(3)
K(1)-O(2)#1 2.980(3)
K(1)-O(3)#1 2.735(3)
K(1)-O(4)#2 2.913(3)
K(1)-O(4)#3 2.913(3)</td><td>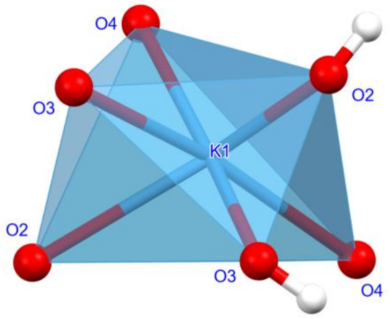

TRIGONAL ANTIPRISM (OC-6)</td></tr>
<tr><td>LK = 7
K(2)-O(1W) 2.709(4)
K(2)-O(2W) 2.750(3)
K(2)-O(1)#8 2.820(2)
K(2)-O(2)#8 2.944(3)
K(2)-O(13)#8 2.859(3)
K(2)-O(3)#9 2.837(3)
K(2)-O(4)#9 3.280(3)</td><td>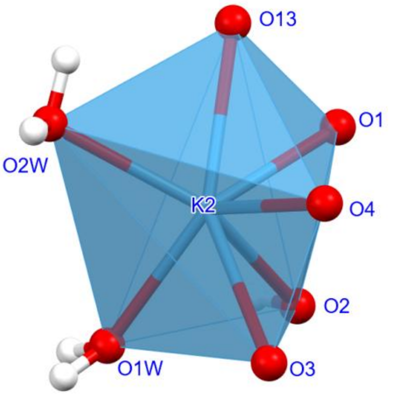

CAPPED OCTAHEDRON (OCF-7)</td></tr>
<tr><td>LK = 6 (on 2-fold axis)
Na(1)-O(5) 2.578(2)
Na(1)-O(6) 2.344(3)
Na(1)-O(4W) 2.273(4)
Na(1)-O(5)#10 2.578(2)
Na(1)-O(6)#10 2.344(3)
Na(1)-O(4W)#10 2.273(4)</td><td>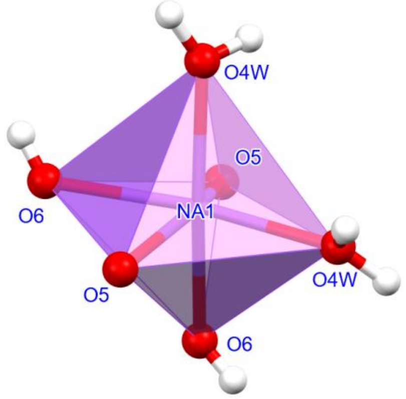

OCTAHEDRON (OC-6)</td></tr>
</table>

Symmetry transformations used to generate equivalent atoms:
#1 -x+1,-y+1,z #2 -x+1,-y+1,z+1 #3 x,y,z+1
#4 -x+1/2,y+1/2,-z #5 x+1/2,-y+1/2,-z #6 x+1/2,-y+1/2,-z+1
#7 -x+1/2,y+1/2,-z+1 #8 -x+1/2,y-1/2,-z #9 x-1/2,-y+1/2,-z
#10 -x+1,-y,z #11 -x+1/2,y-1/2,-z+1 #12 x,y,z-1

Table S14. Cation coordination and hydrogen bonds in the Glc-1P(NH_4)Na crystal

LK = 6

Na(1)-O(5)	2.535(3) Å
Na(1)-O(6)	2.365(6)
Na(1)-O(1W)	2.317(8)
Na(1)-O(5)#1	2.535(3)
Na(1)-O(6)#1	2.365(6)
Na(1)-O(1W)#1	2.317(8)

Symmetry transformations used to generate equivalent atoms: #1 -x+1,-y,z

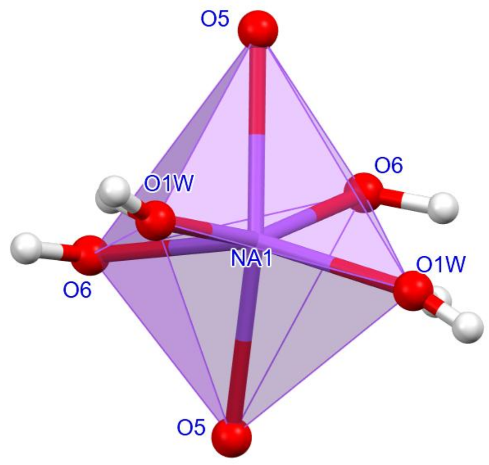

OCTAHEDRON

D-H...A	d(D-H) (Å)	d(H...A) (Å)	d(D...A) (Å)	<(DHA) (º)
N(1)-H(1N1)...O(12)	1.02	1.90	2.782(6)	143
N(1)-H(2N1)...O(11)#2	1.01	2.02	2.817(6)	134
N(1)-H(3N1)...O(3)#3	1.01	2.10	2.782(6)	123
N(2)-H(1N2)...O(2)	1.00	2.18	3.091(5)	151
N(2)-H(2N2)...O(4)#4	1.02	1.90	2.893(5)	166
O(2)-H(2O)...O(11)#2	0.99	1.82	2.780(6)	161
O(3)-H(3O)...O(4W)	0.98	2.13	3.007(8)	148
O(4)-H(4O)...O(12)#5	0.99	1.63	2.611(5)	169
O(6)-H(6O)...O(13)#5	0.99	1.77	2.675(5)	151
O(1W)-H(2W1)...O(2W)#2	0.81	1.91	2.434(16)	122
O(1W)-H(1W1)...O(3W)#6	0.77	2.48	3.069(15)	135
O(2W)-H(1W2)...O(6)#1	0.99	1.80	2.753(12)	161
O(2W)-H(2W2)...O(5)	0.99	2.65	3.205(12)	116
O(2W)-H(2W2)...O(11)	0.99	1.79	2.473(13)	123
O(3W)-H(1W3)...O(13)	0.95	1.76	2.690(7)	167
O(3W)-H(2W3)...O(12)#7	1.01	1.82	2.761(7)	154
O(4W)-H(1W4)...O(3W)#4	1.04	1.86	2.823(9)	152
O(4W)-H(2W4)...O(13)#8	0.86	2.06	2.883(7)	160

Symmetry transformations used to generate equivalent atoms:
#1 -x+1,-y,z #2 x,y,z+1 #3 x-1/2,-y+1/2,-z+2
#4 -x+1,-y+1,z+1 #5 x+1/2,-y+1/2,-z+1 #6 -x+1/2,y-1/2,-z+1
#7 -x+1/2,y+1/2,-z+1 #8 -x+1,-y+1,z

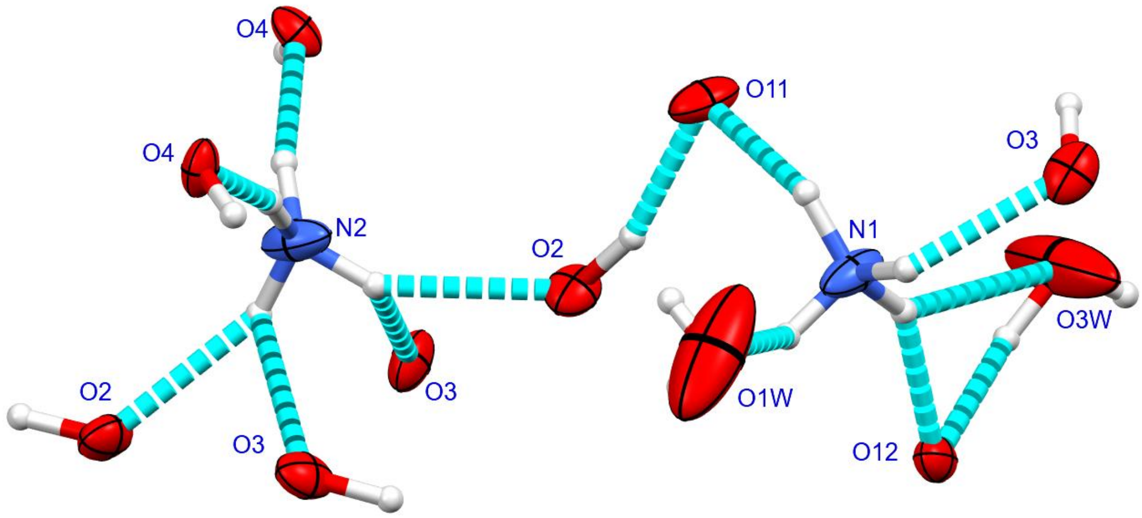

Ammonium cation….. water/hydroxyl interactions

Table S15. Cation coordination in the Glc-1PH(NH4)K crystal. The K cation occupancy is 31%

**LK = 8**		
K(1)-O(3)#1	2.725(10)	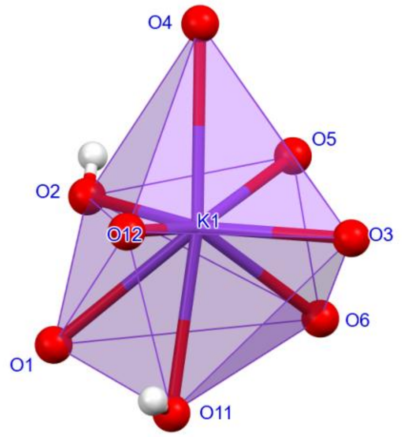
K(1)-O(2)	2.725(10)	
K(1)-O(6)#2	2.857(9)	
K(1)-O(5)#2	2.908(9)	
K(1)-O(12)#3	3.017(9)	
K(1)-O(11)	3.026(10)	
K(1)-O(1)	3.125(10)	
K(1)-O(4)#4	3.274(10)	
		BICAPPED TETRAGONAL PRISM

Symmetry transformations used to generate equivalent atoms:
#1 -x+1,y+1/2,-z+1/2 #2 -x+3/2,-y+1,z-1/2
#3 x-1/2,-y+3/2,-z+1 #4 -x+1/2,-y+1,z-1/2

Hydrogen bonds O-H…O

D-H...A	d(D-H)	d(H...A)	d(D...A)	<(DHA)
O(2)-H(2O)...O(12)#2	0.82	1.90	2.698(3)	166
O(3)-H(3O)...O(6)#9	0.82	2.42	2.927(3)	121
O(4)-H(4O)...O(13)#10	0.82	1.94	2.751(3)	169
O(6)-H(6O)...O(12)#11	0.82	1.95	2.764(3)	171
O(11)-H(11)...O(13)#3	0.82	1.77	2.564(3)	162

Symmetry transformations used to generate equivalent atoms:
#2 -x+3/2,-y+1,z-1/2 #3 x-1/2,-y+3/2,-z+1
#9 x-1/2,-y+1/2,-z+1 #10 x-1,y,z
#11 -x+1,y-1/2,-z+3/2 #12 x+1,y,z

Table S16. Hydrogen bonds in the Glc-1P2(NH4) crystal

NH_4^+…..O distances

N(1)...O(1)	2.973(4)	**N(2) – on 2-fold axis**	
N(1)...O(2)	3.016(4)	N(2)...O(2)	3.013(3)
N(1)...O(13)	2.906(4)	N(2)...O(4)#5	2.937(4)
N(1)...O(3)#1	2.949(4)	**N(3) - disordered**	
N(1)...O(4)#1	3.327(4)	N(3)...O(5)	2.806(12)
N(1)...O(2W)#3	3.093(6)	N(3)...O(5)#2	3.099(11)
N(1)...O(3WA)#4	2.850(10)	N(3)...O(6)#2	2.759(19)
N(1)...O(3WB)#4	2.691(10)	N(3)...O(2W)#5	2.940(12)

Geometry of hydrogen bonds

Donor acceptor	N-H (Å)	H….O (Å)	N…O (Å)	N-H…O (^{o})
N(1)-H(1N1)...O(2W)#3	0.96	2.23	3.093(6)	149
N(1)-H(2N1)...O(1)	0.96	2.29	2.973(4)	128
N(1)-H(2N1)...O(13)	0.96	2.16	2.906(4)	134
N(1)-H(3N1)...O(3WA)#4	0.95	2.18	2.850(10)	126
N(1)-H(3N1)...O(3WB)#4	0.95	1.82	2.691(10)	151
N(1)-H(3N1)...O(2)	0.95	2.52	3.016(4)	112
N(1)-H(4N1)...O(3)#1	0.94	2.40	2.949(4)	117
N(1)-H(4N1)...O(4)#1	0.94	2.46	3.327(4)	152
N(2)-H(1N2)...O(4)#5	0.95	2.00	2.937(4)	167
N(2)-H(2N2)...O(2)	0.95	2.07	3.013(3)	172
N(3)-H(1N3)...O(5)	1.02	1.85	2.806(12)	154
N(3)-H(2N3)...O(2W)#5	0.98	2.09	2.940(12)	144
N(3)-H(4N3)...O(5)#2	0.90	2.27	3.099(11)	153
N(3)-H(4N3)...O(6)#2	0.90	2.13	2.759(19)	126

Symmetry transformations used to generate equivalent atoms:

#1 -x,-y+1,z #2 -x+1,-y+1,z #3 x-1/2,-y+1/2,-z+1

#4 x-1/2,-y+1/2,-z+2 #5 x,y,z+1 #6 x+1/2,-y+1/2,-z+1

#7 -x+1/2,y+1/2,-z+2 #8 -x+1/2,y+1/2,-z+1
